# A minimal model for the role of Rim4 in regulating meiotic exit in budding yeast

**DOI:** 10.1091/mbc.E25-08-0379

**Published:** 2026-04-06

**Authors:** V. Abigail Marquez Davila, Pallavi Gadgil, Anna Zike, Gisela Cairo, Renyu Wang, Sima Setayeshgar, Soni Lacefield

**Affiliations:** ^a^Department of Physics, Indiana University, Bloomington, IN; ^b^Department of Biochemistry and Cell Biology, Geisel School of Medicine at Dartmouth, Hanover, NH; ^c^Department of Biology, Indiana University, Bloomington, IN; University of California, Santa Cruz

## Abstract

Meiosis ensures the formation of haploid gametes through two rounds of chromosome segregation after one round of DNA replication. How this cell-cycle process is restricted to two, and only two divisions, is poorly understood. In budding yeast, RNA-binding protein Rim4 binds various mRNAs to prevent their translation. At the onset of meiosis II, phosphorylation and degradation of Rim4, along with the concomitant release of sequestered mRNA, play an important role in ensuring meiotic exit. Building on previous work, we developed a parsimonious mathematical model of meiotic termination that elucidates the role of Rim4–mRNA release and translation of *AMA1* mRNA in the fidelity of meiotic exit. Central to our model is the accumulation of Ama1 protein, a meiosis-specific activator of APC/C. Our mathematical model predicted further outcomes, which we tested experimentally. We found that either slowing Rim4 degradation or disrupting APC/C^Ama1^ activity delayed meiosis II. In some cells, this disruption prevented meiotic exit entirely, leading them to re-enter cell-cycle oscillations after meiosis II. These findings demonstrate that the timely activation of this regulatory network is crucial for ensuring irreversible meiotic exit.

## Abstract (executive summary)

- Meiosis is uniquely regulated to ensure two rounds of chromosome segregation after one round of DNA replication. Limiting meiosis to two and only two divisions is important for the production of viable gametes, yet the precise regulatory network governing meiotic termination is not fully understood.
- We sought to further understand the minimal components required for meiotic exit by using mathematical modeling combined with experimental analysis in budding yeast. We describe a model in which the timely release of mRNAs from the RNA-binding protein Rim4 is important for timely meiotic exit and to prevent cell-cycle oscillations after meiosis II.

## INTRODUCTION

The specialized cell division process of meiosis produces haploid gametes from a diploid progenitor cell. This critical reduction in chromosome number is achieved through a single round of DNA replication followed by two distinct rounds of chromosome segregation in which homologous chromosomes segregate in meiosis I and sister chromatids separate in meiosis II. The transition to exit from meiosis is an irreversible step, essential not just for completing cell division, but also for coordinating gamete differentiation (Neiman, 2011; Winter, 2012; MacKenzie and Lacefield, 2020). A dysregulation of meiotic exit has profound consequences, including the loss of gamete competence.

Irreversibility in cell-cycle transitions often occurs through the proteolytic degradation of major cell-cycle regulators, along with a systems-level feedback network to prevent resynthesis of the protein and to maintain the stable state (King *et al.*, 1996; Reed, 2003; López-Avilés *et al.*, 2009). Mitotic and meiotic exit rely on common machinery, yet there are important differences in the regulatory networks that govern exit. These differences are key for coupling cell-cycle events to the next stages: entering either G_0_ or G_1_ from mitosis, or for ensuring gamete differentiation and competence from meiosis. Budding yeast is a fantastic model organism for studying meiotic exit in comparison with mitotic exit. Experiments combined with mathematical models have revealed the feedback loops required for irreversible mitotic exit (Bosl and Li, 2005; Ciliberto *et al.*, 2005; López-Avilés *et al.*, 2009; Kraikivski *et al.*, 2015; Novak and Tyson, 2022). Whereas, the components and regulatory network important for meiotic exit are currently being defined (Attner and Amon, 2012; Winter, 2012; Berchowitz *et al.*, 2013; 2015; Argüello-Miranda *et al.*, 2017; Carpenter *et al.*, 2018; MacKenzie and Lacefield, 2020; Paulissen *et al.*, 2020; Wang *et al.*, 2020; Oz *et al.*, 2022; Rojas *et al.*, 2023; Seitz *et al.*, 2023).

For both mitosis and meiosis, exit requires the halt of the oscillatory activity of cyclin-dependent kinase, Cdk1. The binding of a B-type cyclin activates Cdk1 to phosphorylate substrates, such as those involved in spindle assembly and attachment of kinetochores to spindle microtubules (Enserink and Kolodner, 2010; MacKenzie and Lacefield, 2020). The meiotic divisions are controlled by two waves of cyclin-dependent kinase activity. During the transition between meiosis I and meiosis II, Cdk1-cyclin B activity declines for anaphase I spindle disassembly but then rises again for metaphase II spindle assembly. For anaphase onset in mitosis and meiosis, Cdk1-cyclin B phosphorylates and activates its inhibitor, the anaphase-promoting complex/cyclosome (APC/C), a ubiquitin ligase that, when bound to its coactivator Cdc20, ubiquitinates and targets cyclin B for proteasomal degradation (Rudner and Murray, 2000; Rudner *et al.*, 2000). The Cdc14 phosphatase is released from the nucleolus and dephosphorylates Cdk1-cyclin B substrates (Visintin *et al.*, 1998; Buonomo *et al.*, 2003; Marston *et al.*, 2003; Kamieniecki *et al.*, 2005; Attner and Amon, 2012; Manzano-López and Monje-Casas, 2020). For cells to exit mitosis and meiosis, cyclin B levels and Cdk1-cyclin B activity must remain low (López-Avilés *et al.*, 2009; Enserink and Kolodner, 2010).

During both mitotic and meiotic exit, cyclin resynthesis is also blocked. Two coactivators of the APC/C are needed for full degradation of the mitotic cyclins: Cdc20 and Cdh1 (Enserink and Kolodner, 2010). APC/C^Cdh1^ becomes active in late mitosis and stays active through G_1_ (Enserink and Kolodner, 2010). APC/C^Cdh1^ also targets Ndd1 for degradation, an activator of the Mcm1-Fkh2 transcription factor that induces expression of the G_2_–M genes, including cyclins Clb1 and Clb2 (Koranda *et al.*, 2000; Kumar *et al.*, 2000; Pic *et al.*, 2000; Darieva *et al.*, 2003; Sajman *et al.*, 2015; Zhu *et al.*, 2023). Additionally, an inhibitor of Cdk1-cyclin B activity maintains the stable state. Therefore, irreversible mitotic exit occurs through the decline in Cdk1-cyclin B activity, the release of Cdc14 phosphatase from the nucleolus to dephosphorylate Cdk1 substrates, the degradation of Ndd1 to prevent resynthesis of the cyclins, and through a feedback loop that activates an inhibitor of Cdk1 (López-Avilés *et al.*, 2009; Enserink and Kolodner, 2010; Sajman *et al.*, 2015; Linke *et al.*, 2017).

For meiotic exit, APC/C^Cdc20^, along with a meiosis-specific coactivator of the APC/C, Ama1, is required for full cyclin B degradation. APC/C^Cdc20^ initiates cyclin B degradation, and then APC/C^Ama1^ further ubiquitinates any remaining cyclin B for degradation in anaphase II (Cooper *et al.*, 2000; Cooper and Strich, 2011). APC/C^Ama1^ has other substrates in addition to those of APC/C^Cdc20^, including the middle meiosis transcription factor Ndt80, other meiotic regulators such as the polo kinase Cdc5, and proteins involved in inhibiting spore formation (Cooper *et al.*, 2000; Oelschlaegel *et al.*, 2005; Penkner *et al.*, 2005; Diamond *et al.*, 2009; Tan *et al.*, 2011; Okaz *et al.*, 2012; Argüello-Miranda *et al.*, 2017; Omerza *et al.*, 2018; Rimal *et al.*, 2020; Oz *et al.*, 2022; Rojas *et al.*, 2023). Ndt80 induces transcription of many genes needed for the meiotic divisions and spore formation, including those that encode the B-type cyclins important for meiosis: *CLB1*, *CLB3*, and *CLB4* (Chu *et al.*, 1998; Chu and Herskowitz, 1998; Hepworth *et al.*, 1998). Therefore, the subsequent proteasomal degradation of cyclins and the transcription factor that induces cyclin gene expression will prevent the accumulation of new cyclins, thereby halting the activity of Cdk1, leading to an irreversible meiotic exit.

Several processes temporally regulate the activity of APC/C^Ama1^ during meiosis. First, although the transcription of *AMA1* is upregulated by Ndt80, translation of the mRNA primarily occurs in meiosis II (Chu and Herskowitz, 1998; Brar *et al.*, 2012; Berchowitz *et al.*, 2013; Cheng *et al.*, 2018). *AMA1* mRNA is thought to be sequestered by the translational repressor Rim4, which assembles into an amyloid-like aggregate (Berchowitz *et al.*, 2013; 2015; Carpenter *et al.*, 2018). Rim4 is heavily phosphorylated and is thought to then release the mRNAs for translation before its subsequent degradation (Berchowitz *et al.*, 2013; 2015; Jin *et al.*, 2015; Carpenter *et al.*, 2018). Second, during meiosis I, APC/C^Ama1^ is inhibited by Polo kinase, Cdc5, specifically when bound to Spo13 (Rojas *et al.*, 2023). Spo13 binds Cdc5 in meiosis I but is then targeted for degradation by APC/C^Cdc20^ at the end of anaphase I. Finally, APC/C^Ama1^ is inhibited by Cdk1-Clb1 (Okaz *et al.*, 2012). Therefore, once *AMA1* mRNAs are released by Rim4 in metaphase II and translated, APC/C^Ama1^ only becomes active after a decline in Cdk1-Clb1 activity when APC/C^Cdc20^ targets Clb1 for degradation. Whether the other B-type cyclins take over this function when Clb1 is absent is currently not known.

The activities of APC/C^Cdc20^, APC/C^Ama1^, and the meiosis-specific Sps1 kinase are important for anaphase II spindle breakdown and meiotic cytokinesis (Argüello-Miranda *et al.*, 2017; Paulissen *et al.*, 2020; Seitz *et al.*, 2023). The casein kinase Hrr25 promotes meiosis II spindle disassembly by activating the degradation of Clb1 through APC/C^Cdc20^ and APC/C^Ama1^ (Argüello-Miranda *et al.*, 2017). Sps1 functions downstream of the Hippo-like kinase Cdc15 for the release of the Cdc14 phosphatase from the nucleolus into the cytoplasm (Paulissen *et al.*, 2020). Once released, Cdc14 dephosphorylates substrates of Cdk1-cyclin B (Visintin *et al.*, 1998; Manzano-López and Monje-Casas, 2020). Cytokinesis in budding yeast meiosis is independent of the contractile actino-myosin ring but instead occurs with the closure of the prospore membrane, which requires both APC/C^Ama1^ and Sps1 activity (Taxis *et al.*, 2006; Diamond *et al.*, 2009; Paulissen *et al.*, 2020; Neiman, 2024).

We sought to further understand the unique meiotic exit regulatory network in budding yeast, focusing on the steps in meiosis II that lead to increased APC/C^Ama1^ activity and the termination of the oscillations of Cdk1-cyclin B. The Rim4 translational repressor is degraded by autophagy and potentially the proteasome (Carpenter *et al.*, 2018; Wang *et al.*, 2020). Our previous work showed that with autophagy inhibition, Rim4 persists, and cells do not exit meiosis. Instead, the autophagy-inhibited cells undergo aberrant rounds of spindle formation, spindle elongation, chromosome segregation, and Cdc14 nucleolar release and return after meiosis II. The mRNAs held by Rim4 are not translated, and the substrates of APC/C^Ama1^ are not degraded. Rim4 may also be degraded by the proteasome (Carpenter *et al.*, 2018). Here, our goal was to understand the network that ensures termination of cell-cycle oscillations after meiosis II. Using experiments and mathematical modeling, we further define the components of a minimal meiotic exit network. Our model demonstrates how the release of the translational repression of Rim4 mRNA targets, including *AMA1*, allows the accumulation and subsequent activation of APC/C^Ama1^, which is crucial for irreversible meiotic exit.

## RESULTS AND DISCUSSION

### Delayed Rim4 clearance results in extra rounds of spindle pole body accumulation and spindle assembly after meiosis II

**FIGURE 1:**
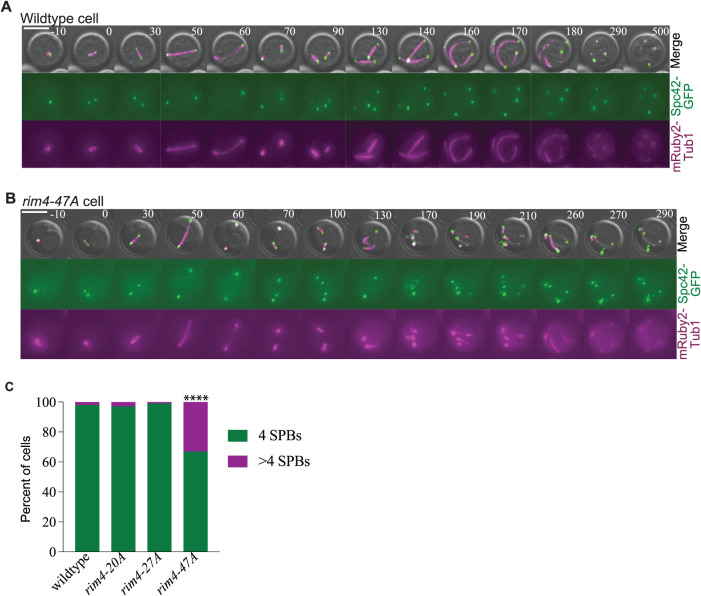
The *rim4-47A* cells undergo extra rounds of SPB accumulation and spindle formation after meiosis II. (A and B) Time-lapse images of a WT (A) and a *rim4-47A* (B) cell undergoing meiosis. Cells express *SPC42-GFP* and *mRuby2-TUB1* to monitor SPBs and spindles, respectively. Images were taken every 10 min; *t* = 0 represents the time at bipolar spindle formation (prometaphase/metaphase I). Not all timepoints shown, but images were chosen to show specific stages of meiosis (A and B) and additional SPB and spindle formation (B). (C) Graph showing the percentage of cells with 4 or >4 SPBs for each genotype. At least 75 cells counted per genotype. Statistical significance with Fisher's exact test *p* < 0.0001.

Rim4 is a key substrate of autophagy, whose degradation has been shown to be important for meiotic exit after meiosis II (Wang *et al.*, 2020). Previous work showed that multisite phosphorylation of the Rim4 C-terminus is needed for normal timing of Rim4 clearance (Carpenter *et al.*, 2018). A mutant version of Rim4 with 47 serine and threonine residues in the C-terminus, *rim4-47A*, resulted in delayed degradation of Rim4 and delayed translation of the Rim4 target *CLB3*. We hypothesized that, like autophagy inhibition, delayed dissociation of Rim4 from mRNA could also cause a failed meiotic exit, leading to additional rounds of spindle pole body (SPB) and spindle accumulation after meiosis II. In support of this hypothesis, extra SPB foci were reported in the original description of *rim4-47A* (Carpenter *et al.*, 2018)*,* but whether this was due to extra rounds of spindle assembly after meiosis II was not clear. To further test our hypothesis, we performed time-lapse microscopy on wild-type (WT) and *rim4-47A* cells. To determine whether cells failed to exit meiosis properly, we monitored WT and *rim4-47A* cells that expressed Green Fluorescent Protein (Spc42-GFP) to monitor SPB accumulation and mRuby2-Tub1 to monitor the spindle. After anaphase II and spindle breakdown, WT cells exited meiosis normally (Figure 1A). In contrast, 33% of *rim4-47A* cells failed to exit meiosis. Instead, they underwent meiosis II spindle breakdown and then initiated additional rounds of SPB accumulation, spindle assembly, and spindle disassembly (Figure 1, B and C). Cells that underwent these additional cell-cycle events accumulated between five and seven SPBs. The variability in the phenotype is likely due to the stochasticity in the network dynamics at the single-cell and population levels, as further discussed below (see also Supplementary Information). These results suggest that delayed clearance of Rim4 can lead to a failure in meiotic exit.

We next tested two other *rim4* phosphorylation mutants, *rim4-27A* and *rim4-20A*. The *rim4-27A* has 27 serine and threonine residues mutated to alanine at the C-terminus from residues 552-718 (Carpenter *et al.*, 2018). The *rim4-20A* has 20 serine and threonine residues mutated to alanine from residues 450 to 552. Together, the two regions with mutations within *rim4-27A* and *rim4-20A* span the total region of the mutated residues in *rim4-47A* (residues 450-718). Furthermore, *rim4-27A* and *rim4-20A* have delayed Clb3 protein accumulation, but not as delayed as the *rim4-47A* mutants, which suggested that a high level of phosphorylation is needed for Rim4–mRNA release (Carpenter *et al.*, 2018). With time-lapse imaging, we found that almost all *rim4-27A* and *rim4-20A* mutants exited meiosis after meiosis II, similar to WT cells (Figure 1C). We conclude that a severe delay in Rim4 degradation and in translation of Rim4 targets prevents cells from exiting meiosis after meiosis II.

### The M-phase cyclin Clb1, but not Clb3, is important for the normal timing of meiosis II

The rapid degradation of Rim4 in metaphase II suggests a model for a switch-like transition into anaphase II: phosphorylation-mediated dissociation of mRNA–Rim4 complexes results in both release of the transcripts to activate meiotic exit pathways, as well as targeting of Rim4 for degradation (Berchowitz *et al.*, 2013; 2015; Carpenter *et al.*, 2018; Wang *et al.*, 2020). Of the known Rim4 target transcripts, two have known or potential roles in the regulation of the transition into meiosis II exit: *CLB3* and *AMA1*(Berchowitz *et al.*, 2013; Carpenter *et al.*, 2018)*.* Although Ama1 has an established role in coupling meiotic exit and spore formation (McDonald *et al.*, 2005; Diamond *et al.*, 2009; Argüello-Miranda *et al.*, 2017; Rimal *et al.*, 2020), the role of Clb3 in meiosis II and meiotic exit is unclear. Previous findings demonstrate that Clb3 binds and activates Cdk1 in meiosis II (Carlile and Amon, 2008), suggesting a model that a sudden burst in Clb3 production and Cdk1-Clb3 activity could drive the cells into anaphase II through the phosphorylation and activation of APC/C^Cdc20^. However, other reports have shown that the *CLB3* deletion does not exhibit a defect in sporulation or tetrad formation (Grandin and Reed, 1993; Dahmann and Futcher, 1995). Therefore, we wanted to further investigate whether Clb3 had a more subtle role in meiosis II, such as in maintaining the appropriate timing of meiosis II.

**FIGURE 2:**
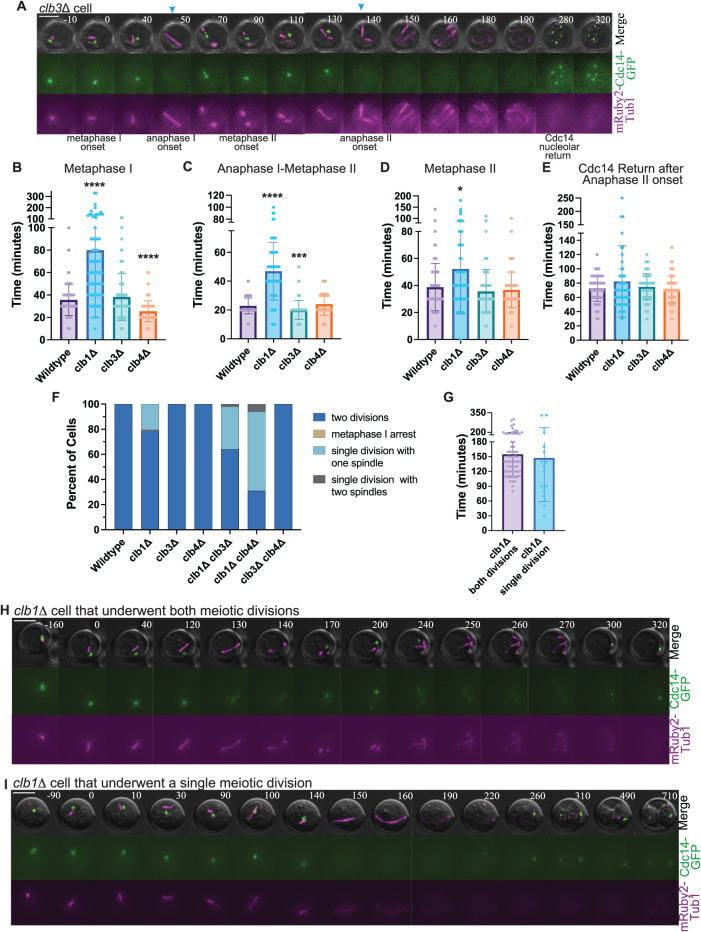
Clb1 is more important than Clb3 and Clb4 in maintaining the normal duration of meiosis II and in ensuring two meiotic divisions. (A) Time-lapse images of a *clb3Δ *cell undergoing meiosis. Cells express *CDC14-GFP* and *mRuby2-TUB1*. Blue arrows show timepoints of Cdc14 nucleolar release in meiosis I and meiosis II. Images were taken every 10 min; *t* = 0 represents the time at bipolar spindle formation (prometaphase/metaphase I). Not all timepoints shown, but images were chosen to show specific stages of meiosis. (B) Graph of the mean time from metaphase I spindle formation to spindle elongation at anaphase I. (C) Graph of mean time from anaphase I spindle elongation to metaphase II spindle formation. (D) Graph of mean time from metaphase II spindle formation to anaphase II spindle elongation. (E) Graph of mean time from Cdc14 nucleolar release to Cdc14 return to the nucleolus during anaphase II. (B−E) *Indicates statistically significant difference from WT (****p < 0.0001, ***p < 0.005, *p < 0.05, Mann–Whitney test, *n* ≥ 75 cells per genotype). (F) Graph comparing the percentage of cells that undergo both meiosis I and meiosis II in the single and double *clb* mutant strains. (G) Graph comparing the time of both divisions in *clb1Δ* cells that undergo two divisions versus the time of the single division in *clb1Δ* cells that undergo one division. (H) Time-lapse images of a *clb1Δ* cell that undergoes two divisions. (I) Time-lapse images of a *clb1Δ* cell that undergoes only one meiotic division.

To this end, we performed time-lapse imaging to measure the duration of the meiotic stages and of meiotic exit in WT and *clb3Δ* cells. To detect the stages of meiosis, we monitored cells expressing mRuby2-Tub1, which marks the spindle (Figure 2A) (Markus *et al.*, 2015). Metaphase II is measured by the duration from the formation of a bipolar spindle to the time of spindle elongation, which initiates anaphase II (Cairo *et al.*, 2022). Although spindle disassembly can be used as a marker of anaphase II duration, mutants that affect meiotic exit disassemble their spindles by breaking down into fragments. Therefore, scoring the timepoint of full meiosis II spindle disassembly is challenging (Argüello-Miranda *et al.*, 2017; Seitz *et al.*, 2023). Instead, we tagged Cdc14 with GFP and scored the time of Cdc14 release and return to the nucleolus in anaphase II (Figure 2A). When released from the nucleolus, the Cdc14 phosphatase dephosphorylates Cdk1 targets (Visintin *et al.*, 1998). The inactivation of polo kinase, Cdc5, allows the return of Cdc14 to the nucleolus (Visintin *et al.*, 2008). Because Cdc5 is a substrate of APC/C^Ama1^, which becomes active at the end of meiosis II and targets Cdc5 for proteasomal degradation, the return of Cdc14 into the nucleolus can also serve as a proxy to identify the timing of APC/C^Ama1^ activity and the initiation of meiotic exit (Okaz *et al.*, 2012; Argüello-Miranda *et al.*, 2017).

The time of metaphase I, anaphase I-to-metaphase II transition, metaphase II, and Cdc14 return to the nucleolus in anaphase II were similar between WT and *clb3Δ* cells (Figure 2, B−E). Given that *CLB3* mRNA binds to Rim4 to delay translation to meiosis II (Carlile and Amon, 2008; Berchowitz *et al.*, 2013), we were surprised by these results: we had hypothesized that the absence of Clb3 and the concomitant loss of the surge in Cdk1-Clb3 activity would result in a slower meiosis II. However, based on our experimental results, we conclude that the loss of *CLB3* does not affect the timings of meiosis II or meiotic exit (Figure 2, D−E).

Given the partial redundancy of cyclins in the cell cycle, we next investigated whether the other B-type cyclins, Clb1 and Clb4, were important for the normal meiosis II timings. We deleted individual cyclins and monitored meiosis. As previously shown in both W303 and SK1 strain backgrounds, the sporulation of *clb4Δ* cells resulted in an ascus with four spores, known as a tetrad. In contrast, sporulation of *clb1Δ* cells resulted in a mixture of cells that underwent one division and two divisions, creating dyads and tetrads, respectively (Dahmann and Futcher, 1995; Rojas *et al.*, 2023) (Figure 2, F−I). The live imaging showed that in our W303 background, 79% of *clb1Δ* cells underwent two divisions, and the remaining 21% underwent only a single meiotic division (Figure 2F). Intriguingly, the timing of the singular *clb1Δ* division was similar to the duration of both divisions in *clb1Δ* cells that undergo two divisions, suggesting that the cells delayed in metaphase I undergo only one division, similar to the findings in the SK1 strain background (Rojas *et al.*, 2023) (Figure 2G). As shown previously, the *clb1Δ* cells were also delayed in meiosis I (Carlile and Amon, 2008; Rojas *et al.*, 2023) and in the transition between meiosis I and meiosis II, whereas *clb4Δ* cells were not delayed (Figure 2, B and C).

Of those *clb1Δ* cells that undergo two divisions, metaphase II was prolonged by ∼15 min compared with WT (Figure 2D). In contrast with *clb1Δ* cells, *clb4Δ* cells had similar timings as WT and *clb3Δ* cells in metaphase II. The time of anaphase II was not significantly different in *clb1Δ* and *clb4Δ* cells compared with WT. The meiosis II delay in *clb1Δ* cells was unexpected because it was previously thought that Cdk1-Clb1 was not active in meiosis II (Carlile and Amon, 2008). Therefore, Clb1 is needed for the normal timing of meiosis II, but the other M-phase cyclins, Clb3 and Clb4, likely compensate for the loss of Cdk1-Clb1 activity.

We wanted to further understand the compensatory mechanisms of the cyclins and used time-lapse microscopy to monitor double mutant combinations of *CLB1*, *CLB3*, and *CLB4*. Previous work showed that *clb1Δ clb3Δ*, and *clb1Δ clb4Δ* double mutants had an increase in dyad spores, suggesting a misregulation of meiosis (Dahmann and Futcher, 1995). With live imaging, we found that 36% of the *clb1Δ clb3Δ* double mutant cells that entered meiosis underwent a single meiotic division with a single round of Cdc14 nucleolar release (Figure 2F). Of the double mutants tested, *clb1Δ clb4Δ* mutants had the most severe phenotype, with 63% of cells undergoing a single division. In contrast, 100% of the *clb3Δ clb4Δ* double mutants, in which Clb1 is the only M-phase cyclin present, underwent both meiotic divisions. Combined, our results suggest that Clb3 and Clb4 contribute to meiotic regulation, but Clb1 alone is sufficient for ensuring that cells undergo both meiotic divisions.

### A minimal mathematical model for the transition from meiosis II to meiotic exit

**FIGURE 3:**
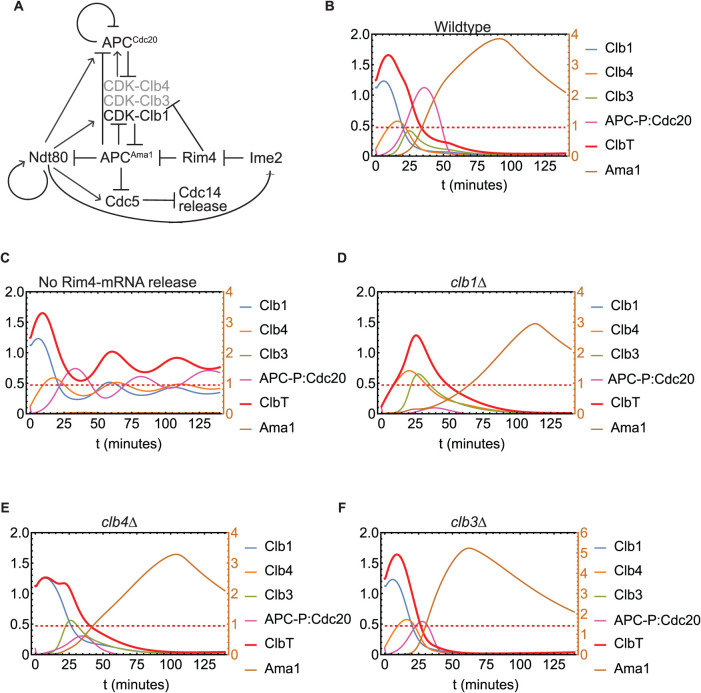
A minimal mathematical model simulates meiotic exit. (A) Wiring diagram showing the minimal regulatory network for meiotic exit on which the mathematical model is based. Dynamics for key network elements in (B) WT cells; (C) the absence of mRNA release from Rim4; (D) *clb1Δ* mutant; (E) *clb4Δ* mutant; (F) *clb3Δ* mutant. See Supplemental Figures S1−S10 for the dynamics of all network variables in B−E, including the variables shown here. (B−E) Concentrations are in arbitrary units (a.u.) on the vertical axes. The horizontal axes denote time since the start of meiosis II. The red dashed line indicates a threshold value assumed in the model for total Cdk1-Clb concentration; decline of Cdk1 activity below this threshold is required for proper chromosome segregation and directing the cell toward the sporulation pathway.

Integrating these findings with our previous work and that of others, we can define a minimal network for understanding the role of Rim4 in regulating meiosis II and meiotic exit (Oelschlaegel *et al.*, 2005; Cooper and Strich, 2011; Tan *et al.*, 2011; Okaz *et al.*, 2012; Berchowitz *et al.*, 2013; 2015; Argüello-Miranda *et al.*, 2017; Carpenter *et al.*, 2018; Rojas *et al.*, 2023) (Figure 3A). The network is initiated in metaphase II when the Ndt80 transcription factor maintains expression of the APC/C coactivator Cdc20, the M-phase cyclins, and the cell-cycle kinases Ime2 and Cdc5 (Chu and Herskowitz, 1998; Oelschlaegel *et al.*, 2005; Okaz *et al.*, 2012). Ndt80 also continues to induce its own expression through a positive feedback loop (Chu and Herskowitz, 1998; Winter, 2012; Tsuchiya *et al.*, 2014). As metaphase II progresses, Rim4 phosphorylation by Ime2 results in dissociation of mRNA from Rim4 and the targeting of Rim4 for autophagic degradation (Berchowitz *et al.*, 2013; Carpenter *et al.*, 2018). The released mRNAs, including *CLB3* and *AMA1,* are then translated, leading to a surge in protein expression.

The activation of APC/C^Ama1^ requires several regulatory steps that occur as the cell transitions from metaphase II to anaphase II. Previous work has shown that APC/C^Ama1^ activity is inhibited by Cdk1-Clb1, so the presence of Ama1 protein will not immediately result in APC/C^Ama1^ activity (Okaz *et al.*, 2012). We have added Cdk1-Clb4 and Cdk1-Clb3 as minor inhibitors of APC/C^Ama1^ based on our findings that most *clb1Δ* cells can undergo both meiotic divisions, suggesting that Clb1, Clb4, and Clb3 have some compensatory functions (Figure 2, B−F).

With increasing Cdk1-Clb activity, APC/C^Cdc20^ is phosphorylated and activated (Rudner and Murray, 2000; Rudner *et al.*, 2000). APC/C^Cdc20^ then targets the cyclins and securin for degradation, resulting in anaphase II onset (Salah and Nasmyth, 2000; MacKenzie and Lacefield, 2020). With decreased Cdk1 activity, APC/C^Ama1^ is activated, leading to meiotic exit by targeting the cyclins, Cdc5, and Ndt80 for degradation (Oelschlaegel *et al.*, 2005; Penkner *et al.*, 2005; Cooper and Strich, 2011; Okaz *et al.*, 2012; Argüello-Miranda *et al.*, 2017). With Ama1 activity, the anaphase II spindle disassembles. The degradation of the Ndt80 transcription factor ultimately results in irreversible meiotic exit because the meiotic regulators are no longer synthesized.

To understand this regulatory network at the systems level and corroborate its predictions with our experiments, we developed a deterministic mathematical model of the wiring diagram (Figure 3A), given by a system of nonlinear ordinary differential equations and associated kinetic parameters (See Supplementary Information 1.1–1.3 for model description, equations, limitations, and Supplemental Tables S1−S10 for governing parameters and initial conditions) (Goldbeter and Koshland, 1981; Goldbeter, 1991; Chen *et al.*, 2000; Tyson and Novak, 2001; Novák and Tyson, 2003, 2008, 2008; Okaz *et al.*, 2012; Qiao *et al.*, 2016; Tyson and Novák, 2022). The model describes the behavior of WT cells undergoing meiotic exit during meiosis II (see Supplementary Information; Figure 3B; Supplemental Figure S1). We refer to the model as minimal, given the focus on the role of Rim4 in regulating meiotic exit. Because the B-type cyclins (Clbs) have a compensatory role, we define the duration of metaphase II as the time for the total cyclin levels to drop below a threshold. A single threshold level, given by ∼one-third of the total Cdk1-Clb peak value in WT (in arbitrary units), determined by trial and error, can be used to qualitatively explain WT and all *clb* mutant phenotypes with respect to meiotic exit (Figure 3, B−F; Supplemental Figures S1−S10 and S12). The total cyclin levels initially decline with activation of APC/C^Cdc20^ and then further decline with activation of APC/C^Ama1^. The cyclins do not return after APC/C^Ama1^ activation because the Ndt80 transcription factor is also targeted for degradation.

In our previous experiments, we found that the inhibition of autophagy results in a failure of meiotic exit (Wang *et al.*, 2020). Instead, additional rounds of SPB duplication, spindle elongation, and chromosome segregation ensue. We noted that these oscillations resemble a meiosis II division because chromosome segregation occurred without intervening DNA replication. However, unlike the transition from meiosis I to meiosis II, only a subset of the SPBs reduplicated. The cells typically increased the number of SPBs one at a time, and most cells underwent between one and three rounds of SPB accumulation and spindle formation, suggesting that the oscillations decay over time.

In the current model, this behavior is recapitulated as damped oscillations in the total Cdk1-Clb concentrations in the exit network dynamics (Figure 3C; Supplemental S10). Previous studies have shown that the negative feedback loop between Cdk1-Clb1 and APC/C^Cdc20^ can lead to oscillations (Milo *et al.*, 2002; Novák and Tyson, 2008; Alon, 2019; Tyson and Novák, 2022). An intermediate step serves to provide a delay between Cdk1-Clb1/4 activation of APC/C^Cdc20^ and APC/C^Cdc20^ inhibition of Cdk1-Clb1/4 (via targeting Clbs for ubiquitination and subsequent degradation). The delay is achieved through Cdk1-Clb1/4 phosphorylation of the APC/C core, which is followed by APC/C activation through binding to Cdc20 (Qiao *et al.*, 2016). Additionally, we allow for APC/C^Cdc20^ autoubiquitination of Cdc20 for its degradation (Robbins and Cross, 2010; Foe *et al.*, 2011).

During damped oscillations, after an initial decline in Clbs, the total Clb level - and therefore Cdk1 activity—undergoes cycles of increases and decreases. Consequently, APC/C^Cdc20^ activity also rises and falls. As the total Cdk1-Clb level approaches the threshold in oscillatory dynamics, a new cell cycle can be initiated. However, whether the total Cdk1-Clb level remains above or below the threshold, the differences between high and low Cdk1-Clb activity may not be sufficient to allow additional rounds of chromosome segregation.

We next tested our model by analyzing the behavior of deletion of *CLB1*, *CLB3,* and *CLB4* genes individually (Figure 3, D−F; Supplemental Figure S1−S4). We then compared the observed phenotype of these strains with the results of the simulated network dynamics (Figures 2, D and E, and 3, D–F). In the *clb1Δ* cells that undergo both divisions, the model recapitulates the observed delay in meiosis II (Figures 2, D and E, and 3D; Supplemental Figure S2). For the *clb4Δ* cells, the normal timing of meiosis II that we observed in the experiments agrees with the computed behavior (Figures 2, D and E, and 3E; Supplemental Figure S3). In the model, *clb3Δ* cells undergo meiosis II somewhat faster, due to the loss of the small burst of Cdk1-Clb3 activity upon release of Clb3 mRNA from Rim4 aggregates. However, this slightly faster timing of meiosis II in the model is within the range of the experimental parameters (Figures 2, D and E, and 3F; Supplemental Figure S4). Therefore, our mathematical model simulates the experimental observations for the cyclin mutants.

**FIGURE 4:**
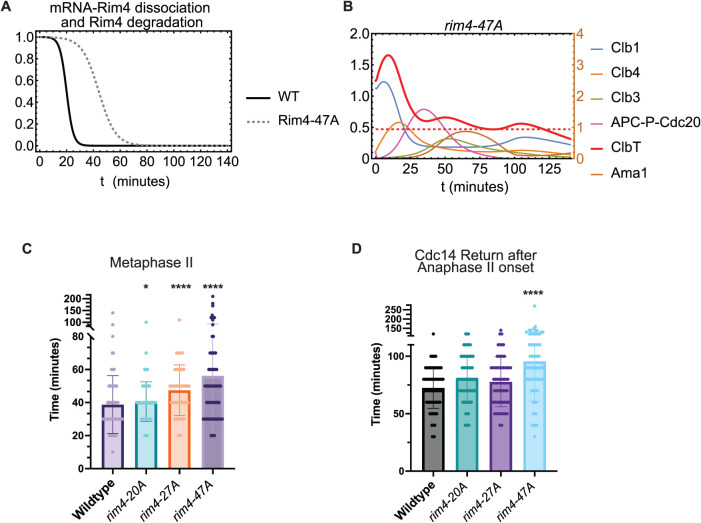
Delaying mRNA–Rim4 dissociation delays meiosis II and results in additional oscillations after anaphase II. (A) Dynamics of mRNA–Rim4 complex dissociation in WT and Rim4 mutant, demonstrating the model assumptions: 1) multisite phosphorylation of Rim4 results in a sigmoidal, or switch-like, dissociation of Rim4–mRNA complex, and resulting in mRNA release for translation and Rim4 clearance 2) mutations in Rim4 phosphorylation sites delay this process (see also Supplemental Figure S11). (B) Network dynamics when mRNA release from Rim4 is delayed, demonstrating damped oscillations in total Cdk1 activity (see also Supplemental Figure S12). (A and B) Concentrations are in arbitrary units (a.u.) on the vertical axes. The horizontal axes denote time since the start of meiosis II. (C) Graph of the mean time from metaphase II spindle formation to anaphase II spindle elongation in different *rim4* mutant strains. (D) Graph of mean time from Cdc14 nucleolar release to Cdc14 return to the nucleolus during anaphase II in *rim4* mutant strains. *Indicates statistically significant difference from WT (****p < 0.0001, **p < 0.007, *p < 0.05, Mann–Whitney test, *n* ≥ 75 cells per genotype).

**FIGURE 5:**
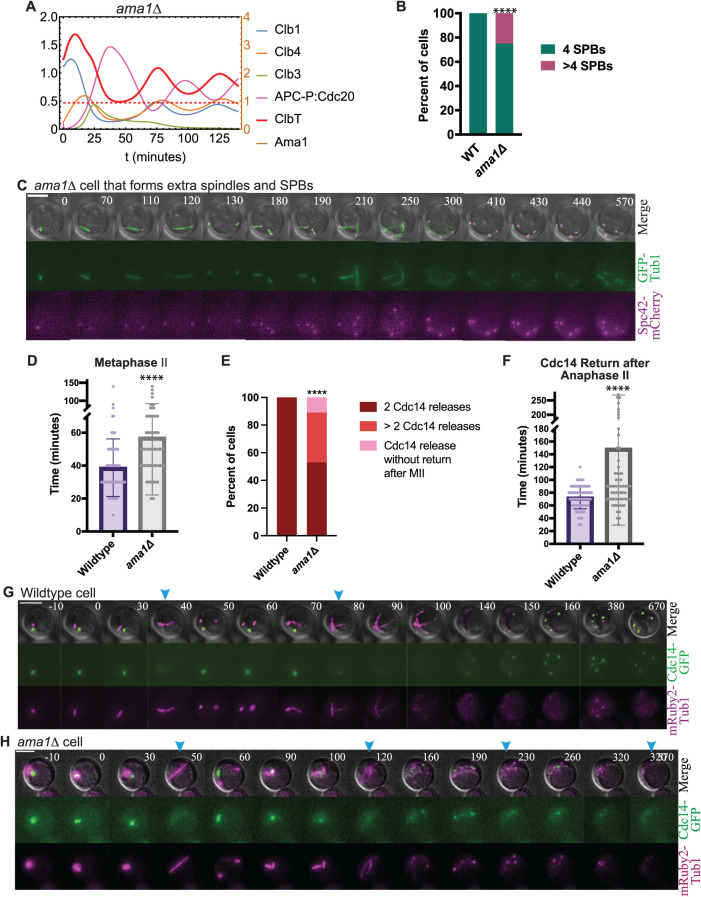
Loss of *AMA1* delays meiosis II and results in additional oscillations after anaphase II. (A) Network dynamics for the *ama1Δ* mutant, demonstrating damped oscillations in total Cdk1 activity analogous to mutants in which mRNA release from Rim4 is delayed or suppressed. Concentrations are in arbitrary units (a.u.) on the vertical axes. The horizontal axes denote time since the start of meiosis II. (B) Graph showing the percentage of cells with 4 or >4 SPBs for each genotype. At least 100 cells counted per genotype. *Indicates statistically significant difference from WT (Fisher's exact test, ****p < 0.0001). (C) Time-lapse images of an *ama1Δ* cell undergoing meiosis. Cells express *SPC42-mCherry* and *GFP-TUB1* to monitor SPBs and spindles, respectively. Images were taken every 10 min; *t* = 0 represents the time at bipolar spindle formation (prometaphase/metaphase I). Not all timepoints are shown, but images were chosen to show specific stages of meiosis. (D) Graph of the mean time from metaphase II spindle formation to anaphase II spindle elongation. *Indicates statistically significant difference from WT (****p < 0.0001, unpaired *t* test with Welch's correction, *n* ≥ 75 cells per genotype) (E) Graph showing the percentage of cells with 2 or >2 Cdc14 releases from the nucleolus. At least 100 cells counted per genotype. *Indicates statistically significant difference from WT (Fisher's exact test, ****p < 0.0001). (F) Graph of mean time from Cdc14 nucleolar release to Cdc14 return to the nucleolus during anaphase II in *rim4* mutant strains. *Indicates statistically significant difference from WT (****p < 0.0001, unpaired *t* test with Welch's correction, *n* ≥ 75 cells per genotype). (G and H) Time-lapse images of a WT cell (G) and an *ama1Δ *cell (H) undergoing meiosis. Cells express *CDC14-GFP* and *mRuby2-TUB1*. Blue arrows show timepoints of Cdc14 nucleolar release in meiosis I and meiosis II. Images were taken every 10 min; *t* = 0 represents the time at bipolar spindle formation (prometaphase/metaphase I). Not all timepoints are shown, but images were chosen to show specific stages of meiosis

In this work, we have adopted a deterministic model and numerical simulation approach to describe the regulatory network in Figure 3A. However, we note that the inherent stochasticity arising from thermal energy of biomolecules gives rise to intrinsic fluctuations in the concentrations of components and rates of reactions constituting biological circuits, thereby constraining the accuracy of biochemical processes (Elowitz *et al.*, 2002; Swain *et al.*, 2002; Ghaemmaghami *et al.*, 2003; Bialek and Setayeshgar, 2005; Raj and Van Oudenaarden, 2008; Brar *et al.*, 2012; Thattai, 2016). A major source of intrinsic noise can be attributed to low copy numbers of mRNA species in gene-protein regulatory networks (Newman *et al.*, 2006). Although past theoretical work points to the universality of Poisson statistics for mRNA dynamics, the resulting distribution of fluctuating protein concentrations depends on many factors, including their function and expression level (Newman *et al.*, 2006; Thattai, 2016). The measured coefficient of variation (given by SD/mean) for protein numbers can be significant, varying between 10 and 40% in yeast (Newman *et al.*, 2006). To address the role of noise in the exit network presented here, a fully or hybrid stochastic implementation is the subject of future work (Gillespie, 1977; 2007; Barik *et al.*, 2010; 2016; Ahmadian *et al.*, 2020). We expect that the decline, followed by threshold crossing of noisy Cdk1-Clb levels in stochastic simulations, will give rise to variability in cell fate outcomes and timing of exit that can be compared with the experimentally observed distributions. For cells exhibiting oscillatory dynamics (Figures 3C, 4B, and 5A), we expect that stochasticity in protein numbers as well as in the biochemical regulation of phosphorylation-mediated mRNA–Rim4 dissociation and Rim4 clearance will lead to population-level heterogeneity in the additional rounds of divisions (4SPBs or >4SPBs).

### Testing predictions of the model: Delayed mRNA–Rim4 dissociation and Rim4 degradation cause a delayed meiosis II and Cdc14 return

Next, we used the mathematical model to guide our experimental analysis. Specifically, we investigated the effect of the timing of Rim4–mRNA complex dissociation and Rim4 clearance on meiotic exit. We describe this process phenomenologically in the model, using a single, switch-like function-of-time 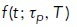, where the parameters 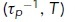, govern the rate and onset of mRNA dissociation (and Rim4 clearance), respectively (Figure 4A; see Supplementary Information, Section 1.1.3). Cooperative models of multisite phosphorylation kinetics could plausibly describe the transition from the mRNA-bound to the mRNA-unbound states of Rim4, mediated by its phosphorylation level (Monod *et al.*, 1965; Koshland *et al.*, 1966; Qian, 2003; Bialek and Setayeshgar, 2008; Salazar and Höfer, 2009; Marzen *et al.*, 2013; Ferrell and Ha, 2014a; 2014b; 2014c). These models display similar sigmoidal functional behavior as the phosphorylation level of Rim4 increases with time, motivating our description according to 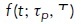. For example, invoking the concerted Monod–Wyman–Changeux model of cooperativity (Monod *et al.*, 1965; Bialek and Setayeshgar, 2008; Marzen *et al.*, 2013), the transition time onset, *T*, is governed by the balance between the intrinsic free-energy bias per cooperative unit, and the ligand discrimination ratio (given by the ratio of dissociation constants for phosphoryl binding to Rim4 in its two—mRNA bound and mRNA unbound—conformational states). Also, as the balance between kinase and phosphatase activity leading to Rim4 phosphorylation increases, this transition time is shortened. Finally, the number of phosphate-binding sites contributes to the sharpness of the transition. We have opted for a phenomenological description according to 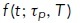, since we are not capturing these mechanistic details in our model. Furthermore, Rim4 clearance is not treated separately in the model and is assumed to be rapid following dissociation of the phosphorylated Rim4–mRNA complex.

In Figure 4A, we show the timing of dissociation of Rim4–mRNA complexes for two cases, representing WT as well as Rim4 mutants in which altered Rim4 phosphorylation kinetics result in a delay in the onset of mRNA dissociation (and Rim4 clearance). In Figure 4B, simulation results demonstrate that whether this onset is strongly delayed with respect to WT, as it is in *rim4-47A* cells, the timing of meiosis II is delayed, and the total cyclin levels initially decline, but then reaccumulate for a possible extra cell cycle. These results are consistent with our experimental data describing extra cell-cycle oscillations in *rim4-47A* cells (Figure 1, B and C).

To further test this prediction experimentally, we analyzed the timing of meiosis II in the strains with mutations in Rim4 phosphorylation sites. Previous work showed that the *rim4-47A* was delayed in meiosis II compared with WT cells (Carpenter *et al.*, 2018). We wanted to expand this analysis to look at anaphase II timing based on Cdc14 release and return. With time-lapse imaging, we measured the duration of metaphase II and anaphase II. We found that *rim4-20A* had similar timings as WT cells (Figure 4, C and D). In contrast, in *rim4-27A* mutants, metaphase II was delayed by an average of 17 min, but anaphase II was not delayed. The *rim4-47A* cells were delayed by ∼20 min in both metaphase II and anaphase II. These results, combined with those from Figure 1, support the predictions from the model that delaying Rim4–mRNA release and subsequent translation slows meiotic exit and allows cell-cycle oscillations to continue after meiosis II (Supplemental Figure S12).

Finally, we also note that degradation of Rim4 is relevant for the dynamics of the exit network only insofar as ensuring that released mRNA is not resequestered. Even if mRNA does not rebind to phosphorylated Rim4, phosphatase activity could result in sufficient dephosphorylation of Rim4 for mRNA rebinding to occur. The present model assumes no rebinding of mRNA (or equivalently, rapid clearance of Rim4). Increasing the delay in the release of mRNA (and clearance of Rim4) leads to later Cdk1-Clb threshold crossing times for Rim4 mutant strains and eventual transition to oscillatory dynamics (Supplemental Figure S12). Table 1 provides the threshold crossing times for Rim4 and other mutant strains from numerical simulations, in agreement with the experimentally measured mean durations of metaphase II. Overall, these results demonstrate the importance of timely Rim4–mRNA complex dissociation and degradation of Rim4 to prevent delays in meiosis II and additional oscillations.

**TABLE 1:** Timing of Cdk1-Clb threshold crossing, 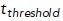
(model), and duration of metaphase II, 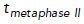 (experiment).

Strain	*t*_threshold_ (min)	*t*_metaphase II_ (min)
WT	34	38 ± 18
*clb1Δ*	48	52 ± 33
*clb4Δ*	40	37 ± 13
*clb3Δ*	27	36 ± 16
*ama1Δ*	45^a^	57 ± 35
*rim4-47A-1*	56	56 ± 37
*rim4-47A-2*	75^a^	—
*rim4-27A*	48	47 ± 15
*rim4-20A*	40	41 ± 12

Note: For each strain, the time at which the total Cdk1-Clb activity in numerical simulations falls below a threshold is compared with the experimentally measured duration of metaphase II. A single threshold value yields consistent results between the model and experiments for the timing of exit for all eight strains. In the model, for WT and Rim4 mutants, the timing and slope of Rim4-mRNA release (and Rim4 degradation) is WT (*T* = 20 min, *τ*_p_^−1^ = 0.2 min⁻¹), rim4-47A-1 (*T* = 38 min, *τ*_p_^−1^ = 0.2 min⁻¹), rim4-47A-2 (*T* = 42 min, *τ*_p_^−1^ = 0.15 min⁻¹), rim4-27A (*T* = 34 min, *τ*_p_^−1^ = 0.2 min⁻¹), and rim4-20A (*T* = 28 min, *τ*_p_^−1^ = 0.2 min⁻¹).

^a^Onset of oscillations

### Testing predictions of the model: loss of Ama1 causes additional rounds of SPB accumulation and spindle formation

Finally, we used the mathematical model to predict how the loss of APC/C^Ama1^ activity would affect the termination of oscillations after meiosis II. APC/C^Ama1^ activity has multiple important roles in meiotic exit, including the targeted degradation of the B-type cyclins and the Ndt80 transcription factor (Cooper *et al.*, 2000; Oelschlaegel *et al.*, 2005; Okaz *et al.*, 2012; Argüello-Miranda *et al.*, 2017; Rojas *et al.*, 2023). To simulate the loss of APC/C^Ama1^ activity, we eliminated Ama1 and found that oscillations occur in total cyclin levels and therefore in Cdk1-Clb activity (Figure 5A; Supplemental Figure S8). Without Ama1, the oscillator module given by Cdk1-Clb and APC/C^Cdc20^ persists, since Clbs are not degraded by APC/C^Ama1^. Whether cells underwent additional cell-cycle oscillations would depend on the total cyclin levels decreasing below and then arising above a threshold. However, whether the oscillations in cyclin levels are not proximal to the threshold but remain above or below it, additional cell-cycle events would not occur.

We tested these predictions of the model with experiments. By monitoring Spc42-GFP and Tub1-mRuby2, we found that 25% of *ama1Δ* cells increased the number of spindles and SPBs after meiosis II (Figure 5, B and C). As previously mentioned, *ama1Δ* cells fragment their spindles during spindle breakdown, making it difficult to assess whether a new shorter spindle is assembled (Argüello-Miranda *et al.*, 2017; Seitz *et al.*, 2023). However, we noticed the acquisition of additional SPBs and short spindles that formed between two SPBs, suggesting that we were observing real spindles and not spindle fragments (Figure 5C). On average, metaphase II was delayed by ∼20 min (Figure 5D).

In WT cells, after Cdc14 is released from the nucleolus in meiosis II, it returns to the nucleolus ∼70 min later (Figure 5, E−G). In contrast, Cdc14 did not return to the nucleolus in 11% of *ama1Δ* cells, and Cdc14 underwent multiple releases in 36% of *ama1Δ* cells (Figure 5, E and H). Furthermore, in the cells in which Cdc14 did return in anaphase II, the timing was delayed on average by 80 min compared with WT (Figure 5F). These results reveal that *ama1Δ* cells can undergo additional cell-cycle oscillations, as shown both by additional spindle assembly and Cdc14 release, as the model predicted. Overall, these results suggest that APC/C^Ama1^ activity in meiosis II is important for meiotic exit by removing both the cyclins and the transcription factor needed for resynthesis of the cyclins.

## CONCLUSIONS

Building on previous findings, we have used mathematical modeling and experimental analysis to define a minimal network for the role of Rim4 in regulating meiosis II exit in budding yeast (Oelschlaegel *et al.*, 2005; Okaz *et al.*, 2012; Berchowitz *et al.*, 2013; 2015; Tsuchiya *et al.*, 2014; Argüello-Miranda *et al.*, 2017; Carpenter *et al.*, 2018; Wang *et al.*, 2020; Rojas *et al.*, 2023). Central to our model is the ultimate activation of APC/C^Ama1^ to allow for the degradation of the network components required for cell-cycle oscillations and the transcription factor needed for resynthesis of those components. Our model describes a complex pathway involving the release of mRNA from the Rim4 translational repressor, the subsequent degradation of Rim4, and the translation of released mRNAs, including *AMA1*. With the accumulation of the Ama1 protein, APC/C^Ama1^ activity is initially inhibited in meiosis II by Cdk1-Clb1 and Cdk1-Clb4. Once APC/C^Cdc20^ becomes active and targets the cyclins for ubiquitination and subsequent proteasomal degradation, APC/C^Ama1^ is activated and then further targets the cyclins, Ndt80, and Cdc5 for proteasomal degradation. Finally, our mathematical model predicted further outcomes that we then tested experimentally to find that with 1) delayed or suppressed release of mRNA from Rim4 and Rim4 clearance or 2) loss of APC/C^Ama1^ activity, meiosis II is delayed, and some cells have a failure in meiotic exit, such that cell-cycle oscillations occur after meiosis II. Our results demonstrate that the activation of this minimal regulatory network is critical for the establishment of irreversible meiotic exit.

Both mitotic and meiotic exit in budding yeast rely on a similar framework centered on the proteasomal degradation of cyclin B and other key cell-cycle regulators. In mitosis, cyclin B is sequentially targeted for degradation by APC/C^Cdc20^ and then APC/C^Cdh1^, leading to a progressive decline in Cdk1 activity (Enserink and Kolodner, 2010). In meiosis, cyclin B is likewise targeted for degradation by APC/C^Cdc20^ and APC/C^Ama1^ (MacKenzie and Lacefield, 2020). Additional regulatory proteins, including the polo-like kinase Cdc5, are targeted by APC/C^Cdh1^ in mitosis and APC/C^Ama1^ in meiosis (Visintin *et al.*, 2008; Okaz *et al.*, 2012). In both cell cycles, the drop in Cdk1 activity prevents further substrate phosphorylation, while the release of the Cdc14 phosphatase counteracts prior Cdk1-dependent phosphorylation, promoting exit (Enserink and Kolodner, 2010; MacKenzie and Lacefield, 2020).

To ensure that exit is irreversible, both mitotic and meiotic cells block the feedback loops required for resynthesis of cyclins and other cell-cycle regulators. In mitosis, APC/C^Cdh1^ targets the coactivator Ndd1, thereby inactivating the G_2_–M transcription complex Fkh2-Mcm1 and shutting down cyclin B transcription (Koranda *et al.*, 2000; Kumar *et al.*, 2000; Pic *et al.*, 2000; Darieva *et al.*, 2003; Sajman *et al.*, 2015; Zhu *et al.*, 2023). Additionally, feedback loops ensure the maintenance of the G_1_ state through the production of a Cdk1 inhibitor (López-Avilés *et al.*, 2009; Enserink and Kolodner, 2010). In meiosis, APC/C^Ama1^ targets the Ndt80 transcription factor, which is required for the transcription of the middle meiotic genes, including *CLB1, CLB3*, and *CLB4* (Chu and Herskowitz, 1998; Okaz *et al.*, 2012). Further regulatory networks and the activity of APC/C^Ama1^ ensure the coupling of meiotic exit to spore formation (McDonald *et al.*, 2005; Diamond *et al.*, 2009; Omerza *et al.*, 2018; Rimal *et al.*, 2020; Oz *et al.*, 2022; Seitz *et al.*, 2023). Altogether, the regulatory mechanisms that promote degradation, phosphorylation reversal, and transcriptional shutdown form a robust system that drives the irreversibility of exit.

## MATERIALS AND METHODS

### Growth conditions

For sporulation, strains were grown in synthetic complete medium (1xSC; 0.67% yeast nitrogen base without amino acids; 0.2% dropout mix with all amino acids; and 2% glucose) at 30°C. Cells were then diluted 1:25 into synthetic complete acetate medium (1xSCA; 0.67% yeast nitrogen base without amino acids; 0.2% dropout mix with all amino acids; and 2% acetate) at 30°C for 12 to 16 h. Finally, cells were washed twice in water and resuspended in 1% potassium acetate, and placed on a roller drum at 25°C for 7 to 8 h before imaging.

### Strain construction

The *Saccharomyces cerevisiae* strains used in this study are derivatives of W303 (*ade2-1 his3-11,15 leu2-3,112 trp1-1 ura3-1 can1-100*; Table S12). Genes were targeted for deletions and tagging using standard PCR-based lithium acetate transformation (Janke *et al.*, 2004). The *rim4-47A*, *rim4-27A*, and *rim4-20A* strains were originally obtained from the Berchowitz laboratory, and the C-terminal mutations were transferred into our strain background by PCR amplification and transformation. The mutations were checked via sequencing. Genotypes of transformed strains were verified by PCR or by sequencing. Integrating plasmids *yomRuby2-TUB1* (yeast optimized mRuby2) were digested and integrated into the *URA3* locus (Markus *et al.*, 2015).

### Time-lapse imaging of budding yeast

For time-lapse imaging, 200 µL of cells were concentrated and then adhered onto a concanavalin A-coated coverslip (Sigma; 1 mg/ml in PBS) and inside a homemade chamber (Cairo *et al.*, 2022). To make a monolayer of cells, we spread the cells using a 5% agar plug (made with 1% potassium acetate) and then left the plug on the cells for 12 min to allow time for the cells to adhere to the concanavalin A. 2 mL of the remaining preconditioned 1% potassium acetate was added dropwise to the chamber to float the agar plug. The plug was then removed, and the chamber was put on the microscope immediately for imaging. All meiosis movies were initiated ∼8 h after the introduction of 1% potassium acetate, when the majority of cells were in prophase I.

Cells were imaged on a Nikon Ti2 Eclipse microscope equipped with an Orca-fusion BT digital c-mos camera (Hamamatsu) and a 60x or 100x oil-immersion objective lens. Images were acquired with GFP and Ruby filters with an exposure time of 20 to 40 ms and with neutral density filters transmitting 2 to 5% of light intensity. Five z-stacks at 1.2 µM z-stacks were acquired every 10 min for 12 to 14 h. Brightfield images were acquired at 70 ms at 5% neutral density. For image analysis, z-stacks were combined in a maximum intensity projection in NIS Elements software (Nikon). Fiji software (NIH) was used to create final images with adjustment of brightness and contrast.

### Statistical analysis

Statistical analysis was performed in GraphPad Prism. For meiotic timings, an unpaired, nonparametric Mann–Whitney test with computation of two-tailed exact *P* values was used. The two-sided Fisher's exact test was used to analyze the percentage of cells with additional SPBs.

### Mathematical Modeling

We numerically simulated a parsimonious mathematical model of the wiring diagram in Figure 3A, given by a system of nonlinear ordinary differential equations (Supplementary Information, Eqs. S3–S14), using Python, corroborated by Mathematica's NDSolve function using a stiff solver (Wolfram Research, Inc., Mathematica, Version 13.3, Champaign, IL [2023]). Detailed description of the model, including limitations and future work, is given in the Supplementary Information, and numerical values for all parameters and initial conditions are given in Supplemental Tables S1−S10. The code used for the computational modeling described in this study is publicly available at https://github.com/abimarquez1211/Meiotic-Exit-Code.

## Supporting information





## Abbreviations used:

APC/C: anaphase promoting complex/cyclosome
CLBs: B-type cyclins
CDK: cyclin-dependent kinase
h: hour(s)
GFP: green fluorescent protein
min: minutes
mL: milliliter
mM: millimeter
ms: millisecond
SC: synthetic complete
SCA: synthetic complete with potassium acetate
SPB: spindle pole body
µM: micrometer
µL: microliter
WT: wild-type.

## References

[B1] Ahmadian M, Tyson JJ, Peccoud J, Cao Y (2020). A hybrid stochastic model of the budding yeast cell cycle. NPJ Syst Biol Appl 6, 7. 32221305 10.1038/s41540-020-0126-z PMC7101447

[B2] Alon U (2019). An Introduction to Systems Biology: Design Principles of Biological Circuits. Boca Raton, FL: Chapman and Hall/CRC Press, Taylor & Francis.

[B3] Argüello-Miranda O, Zagoriy I, Mengoli V, Rojas J, Jonak K, Oz T, Graf P, Zachariae W (2017). Casein kinase 1 coordinates cohesin cleavage, gametogenesis, and exit from M phase in meiosis II. Dev Cell 40, 37–52. 28017619 10.1016/j.devcel.2016.11.021

[B4] Attner MA, Amon A (2012). Control of the mitotic exit network during meiosis. Mol Biol Cell 23, 3122–3132. 22718910 10.1091/mbc.E12-03-0235 PMC3418307

[B5] Barik D, Ball DA, Peccoud J, Tyson JJ (2016). A stochastic model of the yeast cell-cycle reveals roles for feedback regulation in limiting cellular variability. PLoS Comput Biol 12, e1005230. 27935947 10.1371/journal.pcbi.1005230 PMC5147779

[B6] Barik D, Baumann WT, Paul MR, Novak B, Tyson JJ (2010). A model of yeast cell-cycle regulation based on multisite phosphorylation. Mol Syst Biol 6, 405. 20739927 10.1038/msb.2010.55 PMC2947364

[B7] Berchowitz LE, Gajadhar AS, van Werven FJ, De Rosa AA, Samoylova ML, Brar GA, Xu Y, Xiao C, Futcher B, Weissman JS, *et al.* (2013). A developmentally regulated translational control pathway establishes the meiotic chromosome segregation pattern. Genes Dev 27, 2147–2163. 24115771 10.1101/gad.224253.113 PMC3850098

[B8] Berchowitz LE, Kabachinski G, Walker MR, Carlile TM, Gilbert WV, Schwartz TU, Amon A (2015). Regulated formation of an amyloid-like translational repressor governs gametogenesis. Cell 163, 406–418. 26411291 10.1016/j.cell.2015.08.060 PMC4600466

[B9] Bialek W, Setayeshgar S (2005). Physical limits to biochemical signaling. Proc Natl Acad Sci USA 102, 10040–10045. 16006514 10.1073/pnas.0504321102 PMC1177398

[B10] Bialek W, Setayeshgar S (2008). Cooperativity, sensitivity, and noise in biochemical signaling. Phys Rev Lett 100, 258101. 18643705 10.1103/PhysRevLett.100.258101

[B11] Bosl WJ, Li R (2005). Mitotic-exit control as an evolved complex system. Cell 121, 325–333. 15882616 10.1016/j.cell.2005.04.006

[B12] Brar GA, Yassour M, Friedman N, Regev A, Ingolia NT, Weissman JS (2012). High-resolution view of the yeast meiotic program revealed by ribosome profiling. Science 335, 552–557. 22194413 10.1126/science.1215110 PMC3414261

[B13] Buonomo SBC, Rabitsch KP, Fuchs J, Gruber S, Sullivan M, Uhlmann F, Petronczki M, Tóth A, Nasmyth K (2003). Division of the nucleolus and its release of CDC14 during anaphase of meiosis I depends on separase, SPO12, and SLK19. Dev Cell 4, 727–739. 12737807 10.1016/s1534-5807(03)00129-1

[B14] Cairo G, MacKenzie A, Tsuchiya D, Lacefield S (2022). Use of time-lapse microscopy and stage-specific nuclear depletion of proteins to study meiosis in *S. Cerevisiae*. J Vis Exp 10.3791/64580 PMC10114469 36314815

[B15] Carlile TM, Amon A (2008). Meiosis I is established through division-specific translational control of a cyclin. Cell 133, 280–291. 18423199 10.1016/j.cell.2008.02.032 PMC2396536

[B16] Carpenter K, Bell RB, Yunus J, Amon A, Berchowitz LE (2018). Phosphorylation-mediated clearance of amyloid-like assemblies in meiosis. Dev Cell 45, 392–405.e6. 29738715 10.1016/j.devcel.2018.04.001 PMC5944619

[B17] Chen KC, Csikasz-Nagy A, Gyorffy B, Val J, Novak B, Tyson JJ (2000). Kinetic analysis of a molecular model of the budding yeast cell cycle. Mol Biol Cell 11, 369–391. 10637314 10.1091/mbc.11.1.369 PMC14780

[B18] Cheng Z, Otto GM, Powers EN, Keskin A, Mertins P, Carr SA, Jovanovic M, Brar GA (2018). Pervasive, coordinated protein-level changes driven by transcript isoform switching during meiosis. Cell 172, 910–923.e16. 29474919 10.1016/j.cell.2018.01.035 PMC5826577

[B19] Chu S, DeRisi J, Eisen M, Mulholland J, Botstein D, Brown PO, Herskowitz I (1998). The transcriptional program of sporulation in budding yeast. Science 282, 699–705. 9784122 10.1126/science.282.5389.699

[B20] Chu S, Herskowitz I (1998). Gametogenesis in yeast is regulated by a transcriptional cascade dependent on Ndt80. Mol Cell 1, 685–696. 9660952 10.1016/s1097-2765(00)80068-4

[B21] Ciliberto A, Lukács A, Tóth A, TysonÍ JJ, Novak B (2005). Rewiring the exit from mitosis. Cell Cycle 4, 4107–4112. 15970669

[B22] Cooper KF, Mallory MJ, Egeland DB, Jarnik M, Strich R (2000). Ama1p is a meiosis-specific regulator of the anaphase promoting complex/cyclosome in yeast. Proc Natl Acad Sci USA 97, 14548–14553. 11114178 10.1073/pnas.250351297 PMC18956

[B23] Cooper KF, Strich R (2011). Meiotic control of the APC/C: Similarities and differences from mitosis. Cell Div 6, 1–7. 21806783 10.1186/1747-1028-6-16 PMC3162515

[B24] Dahmann C, Futcher B (1995). Specialization of B-type cyclins for mitosis or meiosis in *S. cerevisiae*. Genetics 140, 957–963. 7672594 10.1093/genetics/140.3.957 PMC1206679

[B25] Darieva Z, Pic-Taylor A, Boros J, Spanos A, Geymonat M, Reece RJ, Sedgwick SG, Sharrocks AD, Morgan BA (2003). Cell-cycle–regulated transcription through the FHA domain of Fkh2p and the coactivator Ndd1p. Curr Biol 13, 1740–1745. 14521842 10.1016/j.cub.2003.08.053

[B26] Diamond AE, Park J-S, Inoue I, Tachikawa H, Neiman AM (2009). The anaphase promoting complex targeting subunit Ama1 links meiotic exit to cytokinesis during sporulation in *Saccharomyces cerevisiae*. MBoC 20, 134–145. 18946082 10.1091/mbc.E08-06-0615 PMC2613089

[B27] Elowitz MB, Levine AJ, Siggia ED, Swain PS (2002). Stochastic gene expression in a single cell. Science 297, 1183–1186. 12183631 10.1126/science.1070919

[B28] Enserink JM, Kolodner RD (2010). An overview of Cdk1-controlled targets and processes. Cell Div 5, 11. 20465793 10.1186/1747-1028-5-11 PMC2876151

[B29] Ferrell JE, Ha SH (2014a). Ultrasensitivity part I: Michaelian responses and zero-order ultrasensitivity. Trends Biochem Sci 39, 496–503. 25240485 10.1016/j.tibs.2014.08.003 PMC4214216

[B30] Ferrell JE, Ha SH (2014b). Ultrasensitivity part II: Multisite phosphorylation, stoichiometric inhibitors, and positive feedback. Trends Biochem Sci 39, 556–569. 25440716 10.1016/j.tibs.2014.09.003 PMC4435807

[B31] Ferrell JE, Ha SH (2014c). Ultrasensitivity part III: Cascades, bistable switches, and oscillators. Trends Biochem Sci 39, 612–618. 25456048 10.1016/j.tibs.2014.10.002 PMC4254632

[B32] Foe IT, Foster SA, Cheung SK, DeLuca SZ, Morgan DO, Toczyski DP (2011). Ubiquitination of Cdc20 by the APC occurs through an intramolecular mechanism. Curr Biol 21, 1870–1877. 22079111 10.1016/j.cub.2011.09.051 PMC3430386

[B33] Ghaemmaghami S, Huh W-K, Bower K, Howson RW, Belle A, Dephoure N, O'Shea EK, Weissman JS (2003). Global analysis of protein expression in yeast. Nature 425, 737–741. 14562106 10.1038/nature02046

[B34] Gillespie DT (1977). Exact stochastic simulation of coupled chemical reactions. J Phys Chem 81, 2340–2361.

[B35] Gillespie DT (2007). Stochastic simulation of chemical kinetics. Annu Rev Phys Chem 58, 35–55. 17037977 10.1146/annurev.physchem.58.032806.104637

[B36] Goldbeter A (1991). A minimal cascade model for the mitotic oscillator involving cyclin and cdc2 kinase. Proc Natl Acad Sci USA 88, 9107–9111. 1833774 10.1073/pnas.88.20.9107 PMC52661

[B37] Goldbeter A, Koshland DE (1981). An amplified sensitivity arising from covalent modification in biological systems. Proc Natl Acad Sci USA 78, 6840–6844. 6947258 10.1073/pnas.78.11.6840 PMC349147

[B38] Grandin N, Reed SI (1993). Differential function and expression of *Saccharomyces cerevisiae* B-type cyclins in mitosis and meiosis. Mol Cell Biol 13, 2113–2125. 8455600 10.1128/mcb.13.4.2113-2125.1993 PMC359532

[B39] Hepworth SR, Friesen H, Segall J (1998). NDT80 and the meiotic recombination checkpoint regulate expression of middle sporulation-specific genes in Saccharomyces cerevisiae. Mol Cell Biol 18, 5750–5761. 9742092 10.1128/mcb.18.10.5750 PMC109161

[B40] Janke C, Magiera MM, Rathfelder N, Taxis C, Reber S, Maekawa H, Moreno-Borchart A, Doenges G, Schwob E, Schiebel E, *et al.* (2004). A versatile toolbox for PCR-based tagging of yeast genes: New fluorescent proteins, more markers and promoter substitution cassettes. Yeast 21, 947–962. 15334558 10.1002/yea.1142

[B41] Jin L, Zhang K, Xu Y, Sternglanz R, Neiman AM (2015). Sequestration of mRNAs modulates the timing of translation during meiosis in budding yeast. Mol Cell Biol 35, 3448–3458. 26217015 10.1128/MCB.00189-15 PMC4573713

[B42] Kamieniecki RJ, Liu L, Dawson DS (2005). FEAR but not MEN genes are required for exit from meiosis I. Cell Cycle 4, 1093–1098. 15970684

[B43] King RW, Deshaies RJ, Peters JM, Kirschner MW (1996). How proteolysis drives the cell cycle. Science 274, 1652–1659. 8939846 10.1126/science.274.5293.1652

[B44] Koranda M, Schleiffer A, Endler L, Ammerer G (2000). Forkhead-like transcription factors recruit Ndd1 to the chromatin of G_2_–M-specific promoters. Nature 406, 94–98. 10894549 10.1038/35017589

[B45] Koshland DE, Némethy G, Filmer D (1966). Comparison of experimental binding data and theoretical models in proteins containing subunits. Biochemistry 5, 365–385. 5938952 10.1021/bi00865a047

[B46] Kraikivski P, Chen KC, Laomettachit T, Murali TM, Tyson JJ (2015). From START to FINISH: Computational analysis of cell-cycle control in budding yeast. Npj Syst Biol Appl 1. 10.1038/npjsba.2015.16 PMC5516803 28725464

[B47] Kumar R, Reynolds DM, Shevchenko A, Shevchenko A, Goldstone SD, Dalton S (2000). Forkhead transcription factors, Fkh1p and Fkh2p, collaborate with Mcm1p to control transcription required for M-phase. Curr Biol 10, 896–906. 10959837 10.1016/s0960-9822(00)00618-7

[B48] Linke C, Chasapi A, González-Novo A, Al Sawad I, Tognetti S, Klipp E, Loog M, Krobitsch S, Posas F, Xenarios I, *et al.* (2017). A Clb/Cdk1-mediated regulation of Fkh2 synchronizes CLB expression in the budding yeast cell cycle. Npj Syst Biol Appl 3, 7. 28649434 10.1038/s41540-017-0008-1 PMC5460246

[B49] López-Avilés S, Kapuy O, Novák B, Uhlmann F (2009). Irreversibility of mitotic exit is the consequence of systems-level feedback. Nature 459, 592–595. 19387440 10.1038/nature07984 PMC2817895

[B50] MacKenzie AM, Lacefield S (2020). CDK regulation of meiosis: Lessons from *S. cerevisiae* and *S.* *pombe*. Genes 11, 723. 32610611 10.3390/genes11070723 PMC7397238

[B51] Manzano-López J, Monje-Casas F (2020). The multiple roles of the Cdc14 phosphatase in cell-cycle control. Int J Mol Sci 21, 709. 31973188 10.3390/ijms21030709 PMC7038166

[B52] Markus SM, Omer S, Baranowski K, Lee W-L (2015). Improved plasmids for fluorescent protein tagging of microtubules in *Saccharomyces cerevisiae*. Traffic 16, 773–786. 25711127 10.1111/tra.12276 PMC4795465

[B53] Marston AL, Lee BH, Amon A (2003). The Cdc14 phosphatase and the FEAR network control meiotic spindle disassembly and chromosome segregation. Dev Cell 4, 711–726. 12737806 10.1016/s1534-5807(03)00130-8

[B54] Marzen S, Garcia HG, Phillips R (2013). Statistical mechanics of Monod–Wyman–Changeux (MWC) models. J Mol Biol 425, 1433–1460. 23499654 10.1016/j.jmb.2013.03.013 PMC3786005

[B55] McDonald CM, Cooper KF, Winter E (2005). The Ama1-directed anaphase-promoting complex regulates the Smk1 mitogen-activated protein kinase during meiosis in yeast. Genetics 171, 901–911. 16079231 10.1534/genetics.105.045567 PMC1456836

[B56] Milo R, Shen-Orr S, Itzkovitz S, Kashtan N, Chklovskii D, Alon U (2002). Network motifs: Simple building blocks of complex networks. Science 298, 824–827. 12399590 10.1126/science.298.5594.824

[B57] Monod J, Wyman J, Changeux JP (1965). On the nature of allosteric transitions: A plausible model. J Mol Biol 12, 88–118. 14343300 10.1016/s0022-2836(65)80285-6

[B58] Neiman AM (2011). Sporulation in the budding yeast Saccharomyces cerevisiae. Genetics 189, 737–765. 22084423 10.1534/genetics.111.127126 PMC3213374

[B59] Neiman AM (2024). Membrane and organelle rearrangement during ascospore formation in budding yeast. Microbiol Mol Biol Rev 88, e00013–e00024. 38899894 10.1128/mmbr.00013-24 PMC11426023

[B60] Newman JRS, Ghaemmaghami S, Ihmels J, Breslow DK, Noble M, DeRisi JL, Weissman JS (2006). Single-cell proteomic analysis of *S. cerevisiae* reveals the architecture of biological noise. Nature 441, 840–846. 16699522 10.1038/nature04785

[B61] Novák B, Tyson JJ (2003). Modelling the controls of the eukaryotic cell cycle. Biochem Soc Trans 31, 1526–1529. 14641104 10.1042/bst0311526

[B62] Novák B, Tyson JJ (2008). Design principles of biochemical oscillators. Nat Rev Mol Cell Biol 9, 981–991. 18971947 10.1038/nrm2530 PMC2796343

[B63] Novak B, Tyson JJ (2022). Mitotic kinase oscillation governs the latching of cell-cycle switches. Curr Biol 32, 2780–2785.e2. 35504285 10.1016/j.cub.2022.04.016 PMC9616797

[B64] Oelschlaegel T, Schwickart M, Matos J, Bogdanova A, Camasses A, Havlis J, Shevchenko A, Zachariae W (2005). The yeast APC/C subunit Mnd2 prevents premature sister chromatid separation triggered by the meiosis-specific APC/C-Ama1. Cell 120, 773–788. 15797379 10.1016/j.cell.2005.01.032

[B65] Okaz E, Argüello-Miranda O, Bogdanova A, Vinod PK, Lipp JJ, Markova Z, Zagoriy I, Novak B, Zachariae W (2012). Meiotic prophase requires proteolysis of M phase regulators mediated by the meiosis-specific APC/CAma1. Cell 151, 603–618. 23101628 10.1016/j.cell.2012.08.044

[B66] Omerza G, Tio CW, Philips T, Diamond A, Neiman AM, Winter E (2018). The meiosis-specific Cdc20 family-member Ama1 promotes binding of the Ssp2 activator to the Smk1 MAP kinase. Mol Biol Cell 29, 66–74. 29118076 10.1091/mbc.E17-07-0473 PMC5746067

[B67] Oz T, Mengoli V, Rojas J, Jonak K, Braun M, Zagoriy I, Zachariae W (2022). The Spo13/Meikin pathway confines the onset of gamete differentiation to meiosis II in yeast. EMBO J 41, EMBJ2021109446. 10.15252/embj.2021109446 PMC8844990 35023198

[B68] Paulissen SM, Hunt CA, Seitz BC, Slubowski CJ, Yu Y, Mucelli X, Truong D, Wallis Z, Nguyen HT, Newman-Toledo S, *et al.* (2020). A noncanonical hippo pathway regulates spindle disassembly and cytokinesis during meiosis in *Saccharomyces cerevisiae*. Genetics 216, 447–462. 32788308 10.1534/genetics.120.303584 PMC7536847

[B69] Penkner AM, Prinz S, Ferscha S, Klein F (2005). Mnd2, an essential antagonist of the anaphase-promoting complex during meiotic prophase. Cell 120, 789–801. 15797380 10.1016/j.cell.2005.01.017

[B70] Pic A, Lim F, Ross SJ, Veal EA, Johnson AL, Sultan MRA, West AG, Johnston LH, Sharrocks AD, Morgan BA (2000). The forkhead protein Fkh2 is a component of the yeast cell-cycle transcription factor SFF. EMBO J 19, 3750–3761. 10899128 10.1093/emboj/19.14.3750 PMC313965

[B71] Qian H (2003). Thermodynamic and kinetic analysis of sensitivity amplification in biological signal transduction. Biophys Chem 105, 585–593. 14499920 10.1016/s0301-4622(03)00068-1

[B72] Qiao R, Weissmann F, Yamaguchi M, Brown NG, VanderLinden R, Imre R, Jarvis MA, Brunner MR, Davidson IF, Litos G, *et al.* (2016). Mechanism of APC/CCDC20 activation by mitotic phosphorylation. Proc Natl Acad Sci USA 113, E2570–E2578. 27114510 10.1073/pnas.1604929113 PMC4868491

[B73] Raj A, Van Oudenaarden A (2008). Nature, nurture, or chance: Stochastic gene expression and its consequences. Cell 135, 216–226. 18957198 10.1016/j.cell.2008.09.050 PMC3118044

[B74] Reed SI (2003). Ratchets and clocks: The cell cycle, ubiquitylation, and protein turnover. Nat Rev Mol Cell Biol 4, 855–864. 14625536 10.1038/nrm1246

[B75] Rimal A, Kamdar ZP, Tio CW, Winter E (2020). Isc10, an inhibitor that links the anaphase-promoting complex to a meiosis-specific mitogen-activated protein kinase. Mol Cell Biol 40, e00097–20. 32423992 10.1128/MCB.00097-20 PMC7394827

[B76] Robbins JA, Cross FR (2010). Regulated degradation of the APC coactivator Cdc20. Cell Div 5, 23. 20831816 10.1186/1747-1028-5-23 PMC2949745

[B77] Rojas J, Oz T, Jonak K, Lyzak O, Massaad V, Biriuk O, Zachariae W (2023). Spo13/MEIKIN ensures a two-division meiosis by preventing the activation of APC/CAma1 at meiosis I. EMBO J 42, e114288. 37728253 10.15252/embj.2023114288 PMC10577557

[B78] Rudner AD, Hardwick KG, Murray AW (2000). Cdc28 activates exit from mitosis in budding yeast. J Cell Biol 149, 1361–1376. 10871278 10.1083/jcb.149.7.1361 PMC2175138

[B79] Rudner AD, Murray AW (2000). Phosphorylation by Cdc28 activates the Cdc20-dependent activity of the anaphase-promoting complex. J Cell Biol 149, 1377–1390. 10871279 10.1083/jcb.149.7.1377 PMC2175139

[B80] Sajman J, Zenvirth D, Nitzan M, Margalit H, Simpson-Lavy KJ, Reiss Y, Cohen I, Ravid T, Brandeis M (2015). Degradation of Ndd1 by APC/CCdh1 generates a feed forward loop that times mitotic protein accumulation. Nat Commun 6, 7075. 25959309 10.1038/ncomms8075

[B81] Salah SM, Nasmyth K (2000). Destruction of the securin Pds1p occurs at the onset of anaphase during both meiotic divisions in yeast. Chromosoma 109, 27–34. 10855492 10.1007/s004120050409

[B82] Salazar C, Höfer T (2009). Multisite protein phosphorylation—from molecular mechanisms to kinetic models. FEBS J 276, 3177–3198. 19438722 10.1111/j.1742-4658.2009.07027.x

[B83] Seitz BC, Mucelli X, Majano M, Wallis Z, Dodge AC, Carmona C, Durant M, Maynard S, Huang LS (2023). Meiosis II spindle disassembly requires two distinct pathways. Mol Biol Cell 34, ar98. 37436806 10.1091/mbc.E23-03-0096 PMC10551701

[B84] Swain PS, Elowitz MB, Siggia ED (2002). Intrinsic and extrinsic contributions to stochasticity in gene expression. Proc Natl Acad Sci USA 99, 12795–12800. 12237400 10.1073/pnas.162041399 PMC130539

[B85] Tan GS, Magurno J, Cooper KF (2011). Ama1p-activated anaphase-promoting complex regulates the destruction of Cdc20p during meiosis II. Mol Biol Cell 22, 315–326. 21118994 10.1091/mbc.E10-04-0360 PMC3031463

[B86] Taxis C, Maeder C, Reber S, Rathfelder N, Miura K, Greger K, Stelzer EHK, Knop M (2006). Dynamic organization of the actin cytoskeleton during meiosis and spore formation in budding yeast. Traffic 7, 1628–1642. 17118118 10.1111/j.1600-0854.2006.00496.x

[B87] Thattai M (2016). Universal Poisson statistics of mRNAs with complex decay pathways. Biophys J 110, 301–305. 26743048 10.1016/j.bpj.2015.12.001 PMC4724633

[B88] Tsuchiya D, Yang Y, Lacefield S (2014). Positive feedback of NDT80 expression ensures irreversible meiotic commitment in budding yeast. PLoS Genet 10, e1004398. 24901499 10.1371/journal.pgen.1004398 PMC4046916

[B89] Tyson JJ, Novak B (2001). Regulation of the eukaryotic cell cycle: Molecular antagonism, hysteresis, and irreversible transitions. J Theor Biol 210, 249–263. 11371178 10.1006/jtbi.2001.2293

[B90] Tyson JJ, Novák B (2022). Time-keeping and decision-making in the cell cycle. Interface Focus 12, 20210075. 35860005 10.1098/rsfs.2021.0075 PMC9184962

[B91] Visintin C, Tomson BN, Rahal R, Paulson J, Cohen M, Taunton J, Amon A, Visintin R (2008). APC/C-Cdh1-mediated degradation of the Polo kinase Cdc5 promotes the return of Cdc14 into the nucleolus. Genes Dev 22, 79–90. 18172166 10.1101/gad.1601308 PMC2151016

[B92] Visintin R, Craig K, Hwang ES, Prinz S, Tyers M, Amon A (1998). The phosphatase Cdc14 triggers mitotic exit by reversal of Cdk-dependent phosphorylation. Mol Cell 2, 709–718. 9885559 10.1016/s1097-2765(00)80286-5

[B93] Wang F, Zhang R, Feng W, Tsuchiya D, Ballew O, Li J, Denic V, Lacefield S (2020). Autophagy of an amyloid-like translational repressor regulates meiotic exit. Dev Cell 52, 141–151.e5. 31991104 10.1016/j.devcel.2019.12.017 PMC7138260

[B94] Winter E (2012). The Sum1/Ndt80 transcriptional switch and commitment to meiosis in *Saccharomyces cerevisiae*. Microbiol Mol Biol Rev 76, 1–15. 22390969 10.1128/MMBR.05010-11 PMC3294429

[B95] Zhu W, Ding Y, Meng J, Gu L, Liu W, Li L, Chen H, Wang Y, Li Z, Li C, *et al.* (2023). Reading and writing of mRNA m6A modification orchestrate maternal-to-zygotic transition in mice. Genome Biol 24, 67. 37024923 10.1186/s13059-023-02918-9 PMC10080794

