# Supplementary material for: A minimal model for the role of Rim4 in regulating meiotic exit in budding yeast
Source: Mol Biol Cell. 2026 Apr 6;37(5):br15. doi: 10.1091/mbc.E25-08-0379 (PMC13322200; doi:10.1091/mbc.E25-08-0379)
Supplement: Supplementary file 1 [file mbc-37-br15-s001.pdf]

Supplemental Materials

Molecular Biology of the Cell

Davila *et al*.

1 Supplementary Information: Mathematical Model for Meiotic Exit Network

1.1 Model Description

1.1.1 Network Model

The mathematical model given by the system of nonlinear ordinary differential equations, Eqs. 3-14, describes the wiring diagram in Fig. 3A. This model builds on that of Okaz *et al.* [40] which described the irreversible transition from prophase I to metaphase I. We refer to it as a minimal model for meiotic exit, given the focus on the role of Rim4.

The rate of change of the concentration of each variable with time is described by a nonlinear, ordinary differential equation (ODE) given by the relevant biochemical reaction kinetics, described below. To numerically simulate the equations, we used Python[1], corroborated by Mathematica's `NDSolve` function employing a stiff solver (Wolfram Research, Inc., Mathematica, Version 13.3, Champaign, IL (2023)). Values for shared parameters were based on Okaz *et al.* [40], while values for new parameters were determined in a trial and error process aimed at reproducing the behavior of wild-type and mutants studied in this work, consistent with other works [14, 13, 43, 39, 55, 27, 38, 17]. Rate constants are in units of min^{-1}; concentrations are in arbitrary units (a.u.). Parameter values and initial conditions are reported in Tables S1-10.

The key components of this network are cyclin-dependent kinase (Cdk1) heterodimers, Cdk1-Clb1, Cdk1-Clb3 and Cdk1-Clb4; polo kinase, Cdc5 (with role in Cdc14 release from and re-sequestration to the nucleolus, not accounted for in mathematical model); Ndt80, which transcribes Clb1, Clb3, Clb4, Cdc5, Cdc20, and itself through a positive feedback loop; ubiquitin ligase APC/C (anaphase promoting complex), where APC/C alone is unable to target cyclins for degradation, rather its activation is dependent on the binding of cofactors; Ama1 and Cdc20, activators of APC; Rim4, translational repressor of Ama1 and Clb3 mRNA, which is degraded by autophagy or the proteosome in meiosis II (after being phosphophorylated by Ime2, not accounted for in our mathematical model).

Ndt80 autoregulation has been shown to be required for meiosis II completion in budding yeast [53]. We describe Ndt80 positive feedback by a Hill function, with Hill coefficient $1 < n < 2$, consistent with the existence of 2 binding sites for Ndt80 on the *NDT80* promotor (known as MSE, or middle sporulation element) [63], and half-activation constant, K_{Ndt80}, with value corresponding to more graded activation over the dynamical range of Ndt80 in the model (Eq. 6). We explicitly take into account APC/C activation by Cdc20, following [15, 43] (Eq. 9, see also below). However, in the case of APC/C^{Ama1}, as in [40], we assume that the concentration of APC core is not rate-limiting, and therefore the concentration of APC/C^{Ama1} follows that of Ama1 in our model (Eq. 13). Similarly, it is assumed that Cdk1 subunits are present in excess, and their concentration is not rate-limiting; hence the concentrations of Cdk1-Clb1, Cdk1-Clb3, and Cdk1-Clb4 follow that of the cyclin subunits (Eqs. 3, 4, 5). Synthesis and degradation reactions are approximated by mass action kinetics. Reactions describing activation and inactivation by phosphorylation are described according to zeroth order Goldbeter-Koshland kinetics (Eqs. 8, 9, and Eqs. 12, 13), following previous works [26, 40]. Cdk1-Clb1/Clb4/Clb3 inhibit APC/C^{Ama1} by multisite phosphorylation

[1] Code is available publicly on github at `https://github.com/abimarquez1211/Meiotic-Exit-Code`.

(Eqs. 12). The most active form of Ama1 is unphosphorylated. Phosphorylation reduces but does not completely suppress APC/C$^{\mathrm{Ama1}}$ activity. Therefore, Ama1-dependent proteolysis (Eqs. 3, 4, 5, 10, 11) is proportional to the concentration of active, unphosphorylated Ama1 ([Ama1]) as well as to the total concentration of Ama1 ([Ama1$_{\mathrm{T}}$] = [Ama1-P]+[Ama1]).

Rim4 is phosphorylated by Ime2, leading to its disassembly from amyloid-like aggregates, release of bound mRNAs, and targeting by autophagy for degradation in metaphase II. Importantly, for the network modeled here, this allows Ama1 mRNA which is sequestered by Rim4 at the start of meiosis II to be translated, giving rise to a surge of Ama1 protein (Eq. 13). APC/C$^{\mathrm{Ama1}}$ is held inactive due to Cdk1-Clb1/Clb4 inhibition by phosphorylation (Eq. 12). As Cdk1-Clb1/Clb4 levels rise in metaphase II, they phosphorylate and activate APC/C$^{\mathrm{Cdc20}}$ (Eqs. 8, 9). Active APC/C$^{\mathrm{Cdc20}}$ drives the cells into anaphase II by targeting proteins like Clb1/4 for degradation, decreasing Cdk1-Clb1/Clb4 activity (Eqs. 3, 4, 5). With decreased Cdk1-Clb1/Clb4, APC/C$^{\mathrm{Ama1}}$ becomes active and degrades Ndt80, Cdc5, and more Clb1/Clb4 and Clb3 (Eqs. 3-6, 10-11). Without these regulators, the cell exits meiosis. Clb3, also expressed with release of Clb3 mRNA from Rim4-mRNA aggregates (Eq. 7), extends the duration of APC/C$^{\mathrm{Cdc20}}$ activity, but otherwise does not significantly affect the timing of exit for the chosen numerical values of parameters governing Clb3 kinetics in our current model (motivated by experimental results; see main text). Also, in the present model, Cdk1-Clb4 (and later Cdk1-Clb3), in addition to Cdk1-Clb1, inhibits APC/C$^{\mathrm{Ama1}}$ activity by phosphorylation (Eq. 12). As such, the role of the unknown inhibitor of Ama1 in Okaz *et al.* [40] is played by Clb4.

It has been established that the negative feedback loop between Cdk1-Clb (here, given by Clb1/Clb4/3) and APC/C$^{\mathrm{Cdc20}}$ gives rise to oscillatory dynamics [39, 55, 35, 2]. However, oscillations require an intermediate step, which serves to provide a delay between Cdk1-Clb1/4 activation of APC/C$^{\mathrm{Cdc20}}$ and APC/C$^{\mathrm{Cdc20}}$ inhibition (via ubiquitination) of Cdk1-Clb1/4. In our model, this intermediate step is achieved through Cdk1-Clb1/4 phosphorylation of the APC/C core (Eq. 8), followed by its activation through binding to Cdc20 (Eq. 9) [43]. Additionally, we allow for APC/C$^{\mathrm{Cdc20}}$ degradation of Cdc20 (via self-ubiquitination) (Eq. 7). Further mechanistic details of this feedback loop, which include the inhibitory role of phosophorylation of Cdc20 protein [16, 32], could also be important and are the subjects of future work.

Fig. S1-S7 show the full model dynamics for wild-type and various mutants, corresponding to the manuscript figures for which a subset of the variables were included for clarity.

1.1.2 Cdk1-Clb threshold

With inhibition of autophagy, it has been shown that several rounds of spindle formation, spindle elongation, and chromosome segregation ensue [57]. This is recapitulated as damped oscillations in Cdk1-Clb concentrations in the exit network dynamics. Indeed, in the network model considered here, the Cdk1-Clbs play redundant roles, motivating our consideration of the *total* Cdk1-Clb concentration in the network dynamics. Since the decline in Cdk1-Clb activity is critical for exiting meiosis [33], we consider the time it takes from the start of meiosis II ($t = 0$) for the total Cdk1-Clb concentration level to fall below a threshold, given by approximately one third of the peak total Cdk1-Clb level in WT. This time interval is compared with the experimental results on the duration of metaphase II in WT and mutant strains (Table 1). Although this threshold has been determined by trial and error, we

emphasize that a single threshold value yields results that are consistent with experiments for WT and seven mutant phenotypes.

Finally, despite their redundant roles, if we replace the multiple B-type cyclins with a single variable, ClbT, corresponding to the total Cdk1-Clb concentration, we find that the timing of exit is accelerated and does not agree well with experimental results for the duration of metaphase II (Fig. S8). Therefore, the existence of multiple cyclins may help refine the timing of exit, especially given the release of Clb3 later in meiosis II.

1.1.3 mRNA-Rim4 dissociation and Rim4 clearance

We emphasize that a quantitative description of the spatiotemporally coordinated mechanisms of mRNA sequestration through complex formation with Rim4 amyloid aggregates, followed by Rim4 phosphorylation-mediated release of mRNA and degradation of Rim4 via authophagy [57, 29] is beyond the scope of the current work. Rather, here, we describe the dissociation of Rim4-mRNA complexes and Rim4 clearance phenomenologically, given by a single, sigmoidal function of time, $0 \leq f(t; T, \tau_p) \leq 1$, where $f(t; T, \tau_p) = 1$ corresponds to no protein translation (i.e., mRNA is fully sequestered in Rim4 aggregates) and no Rim4 degradation (no autophagy). Specifically, we use the following functional form:

$$f(t; T, \tau_p) = \frac{1}{2}\left[1 - \tanh\left(\frac{t - T}{\tau_p}\right)\right]. \tag{1}$$

Cooperative models [36, 31, 8, 46, 34, 19, 21, 20, 42] of multisite phosphorylation kinetics applied to Rim4 amyloid aggregates could plausibly describe the transition from the mRNA-bound (and unphosphorylated) to the mRNA-unbound (and phosphorylated) state. These models display similar sigmoidal functional behavior as the phosphorylation level of Rim4 increases with time, motivating our description. For example, in the concerted Monod-Wyman-Changeux model [8] describing the transition between the mRNA bound and mRNA unbound states of Rim4 aggregates mediated by phosphorylation, the transition time onset, T, is governed by the balance between the intrinsic free-energy bias per cooperative unit, and the ligand discrimination ratio (given by the ratio of dissociation constants for phosphoryl binding in the two states). Also the balance between kinase and phosphatase activities of Ime2 and Cdc14, respectively, determines the overall phosphorylation level of Rim4, and can shorten or prolong this transition time. Finally, the number of phosphate binding sites contributes to the sharpness of the transition, τ_p^{-1}. In summary, each of the two control parameters T and τ_p have as input several mechanistic parameters that describe the phosphorylation-mediated Rim4-mRNA complex dissociation. We have opted for a phenomenological description of this process according to $f(t; T, \tau_p)$ since we are not capturing these details in our model.

In Fig. S9, we show the role of the parameter T, which determines the time of onset of Rim4-mRNA dissociation (and Rim4 clearance), and the parameter τ_p, which determines the time scale of Rim4-mRNA dissociation, both of which in principle depend on the details of the cooperative, multisite phosphorylation kinetics, as describe above. In this figure, ($T = 20\,\text{min}, \tau_p^{-1} = 0.2\ \text{min}^{-1}$) (red) corresponds to WT, while for parameters given by ($T = 28\,\text{min}, \tau_p^{-1} = 0.2\ \text{min}^{-1}$) (purple), ($T = 34\,\text{min}, \tau_p^{-1} = 0.2\ \text{min}^{-1}$) (green), and ($T = 38\,\text{min}, \tau_p^{-1} = 0.2\ \text{min}^{-1}$) (purple), mRNA-Rim4 dissociation is delayed with respect to WT. For ($T = 44\,\text{min}, \tau_p^{-1} = 0.08\ \text{min}^{-1}$) (yellow) there is both a delay and slower dissociation.

In Figure S10 we show the numerical solution for the total Cdk1-Clb level as a function of time for each case, demonstrating that as mRNA-Rim4 dissociation is delayed, the threshold crossing occurs later in meiosis II, consistent with the experimental observations of prolonged metaphase II (Fig. 4C), and eventual transition to oscillatory dynamics.

We note that degradation of Rim4 is relevant for the dynamics of the exit network only in so far as ensuring that released mRNA is not re-sequestered. Even if mRNA does not rebind to phosphorylated Rim4, with release of Cdc14 from the nucleolus during anaphase II, its phosphatase activity could result in sufficient dephosphorylation of Rim4 for mRNA rebinding to occur. The present model assumes no rebinding of mRNA (equivalent to rapid clearance of Rim4). Separating the effects of mRNA rebinding and Rim4 clearance on the dynamics of exit is the subject of future work.

The rate at which Clb3 and Ama1 protein are produced from mRNA sequestered by Rim4 is given in terms of Eq. 1 as

$$r_i = k_i' \alpha_i \left(1 - f(t; T, \tau_p)\right) e^{-(t-T)/\tau_{\mathrm{mRNA}}} \tag{2}$$

where i = Clb3 or Ama1. The parameters $(\alpha_{\mathrm{Clb3}}, \alpha_{\mathrm{Ama1}})$ reflect the amount of sequestered mRNA, and $(k'_{\mathrm{Clb3s}}, k'_{\mathrm{Ama1s}})$ are the translation rates. The exponentially decaying function accounts for the finite lifetime of released mRNA. The mRNA lifetime is taken to be $\tau_{mRNA} \approx$ 25 minutes (= average mRNA lifetime in yeast, from [22]).

1.2 Limitations and Future Work

Regulation of mitosis in budding yeast has been the subject of extensive experimental studies and related theoretical modeling, where input of new experimental data has resulted in steady refinement of mathematical models of the underlying cell cycle circuitry [54, 14, 13]. In contrast, mathematical modeling of the regulation of meiosis is less well-characterized [16, 40, 6]. In this work, we have presented a parsimonious model of exit from meiosis II that captures experimental observations in budding yeast ranging from wild-type to 9 mutant phenotypes with a single set of parameter values. We similarly expect further refinements of our model, informed by future experiments.

Key limitations of our current model and possible extensions are discussed below:

1. As described above, we have not attempted to describe the mRNA-Rim4 dynamics mechanistically. Rather, we have addressed the role of Rim4 in regulating exit – through the delay or suppression of release of mRNA – phenomenologically. This component of the network dynamics can be further expanded in the following ways:

   (i) Cooperative models of multisite phosphorylation governing mRNA-Rim4 association and dissociation would provide a principled description, that can be related to RNA-binding properties of Rim4 and its mutants [36, 31, 8, 46, 34].

   (ii) RNA/protein (RNP) bodies are an important class of membrane-less organelles that bind and regulate RNA, thereby ensuring the proper execution of a variety of cellular regulatory processes in a spatially and temporally controlled manner [10, 65, 47, 44, 9]. Increasing evidence suggests that the assembly of RNP bodies can be described in terms of liquid-liquid phase transitions; furthermore, intrinsically disordered protein motifs are commonly found in RNP bodies

[58, 56, 62]. Therefore, should future experiments reveal liquid-liquid phase separation as the mechanism governing the interaction of Rim4 and its mRNA binding partners, the dynamics of phase separation and its role in regulating exit will need to be treated in the theoretical model [61].

2. The inherent stochasticity arising from thermal energy of biomolecules gives rise to intrinsic fluctuations in the concentrations of components and rates of reactions comprising biological circuits, thereby constraining the accuracy of biochemical processes [49, 18, 7, 37, 45, 50, 41]. A major source of intrinsic noise can be attributed to low copy numbers of mRNA molecules in gene-protein regulatory networks [37]. Here, we have adopted a deterministic model and numerical simulation approach to describe the regulatory network in Fig. 3A. Future stochastic simulations will address fluctuations in protein and mRNA numbers [24, 25, 4, 3, 1, 11, 23], allowing comparison of numerical results with experimentally measured variabilities in cell fates and timing of meiotic exit. This will be especially relevant for comparison with our experimentally measured population-level distributions of the timing exit and cell fates in meiosis II, where the decline and threshold crossing of noisy Cdk1-Clbs level will give rise to variability in outcomes for exit. Related to this, recent work has suggested that fluctuations in protein numbers are mitigated by phase-separated compartments, thereby reducing noise and enhancing the robustness of biological systems [48, 30, 64], suggesting a further role for mRNA-Rim4 aggregates.

3. Refinements to the network connectivity that are not treated in the present work include other kinase and phosphatase activities (notably Ime2 and Cdc14, respectively), with possible impact on the biochemical oscillator module (presently given by Cdk1-Clbs and APC/C$^{\mathrm{Cdc20}}$) as well as dynamics of mRNA-Rim4 dissociation mediated by the phosphorylation state of Rim4. Additionally, Clb3 does not appear to play a central role in the dynamics of meiotic exit, despite the fact that its mRNA is a target of Rim4. This surprising experimental finding, reproduced in the mathematical model, may point to other role(s) for Clb3 in meiosis II that are not considered here.

4. Quantitative computational models in systems biology typically involve many free parameters, and directly measuring in vivo or in vitro biochemical parameters is challenging. The difficulty in assigning values of these parameters hinders model development and theoretical analysis. Earlier work has shown that the quantitative behavior of many different classes of nonlinear multiparameter models is more sensitive to changes in certain combinations of parameters than others ("stiff" versus "sloppy" parameter subspaces) [51, 52, 60]. Similar analyses on models of the budding yeast cell cycle [5, 59] have revealed an effective reduction in the number of degrees of freedom of the models. For the minimal meiotic exit model presented here, sensitivity analysis of the model will inform how the combinations of parameters constituting the stiff modes change with different formulations of the cost function and constraints, serving as input to experimental tests of these relevant features controlling the timing and fidelity of exit.

1.3 Model Equations

$$\begin{aligned}\frac{d[\mathrm{Clb1}]}{dt} &= k_{Clb1s} + k_{Clb1s'}[\mathrm{Ndt80}] \\ &- \left(k_{Clb1d} + k_{Clb1d'}[\mathrm{Ama1}] + k_{Clb1d''}[\mathrm{Ama1_T}] + k_{Clb1dCdc20}[\mathrm{APC\text{-}P{:}Cdc20}]\right)[\mathrm{Clb1}], \quad (3)\end{aligned}$$

$$\begin{aligned}\frac{d[\mathrm{Clb4}]}{dt} &= k_{Clb4s} + k_{Clb4s'}[\mathrm{Ndt80}] \\ &- \left(k_{Clb4d} + k_{Clb4d'}[\mathrm{Ama1}] + k_{Clb4d''}[\mathrm{Ama1_T}] + k_{Clb4dCdc20}[\mathrm{APC\text{-}P{:}Cdc20}]\right)[\mathrm{Clb4}], \quad (4)\end{aligned}$$

$$\begin{aligned}\frac{d[\mathrm{Clb3}]}{dt} &= k_{Clb3s} + k_{Clb3s'}(1-f(t))[\mathrm{Ndt80}] \\ &- \left(k_{Clb3d} + k_{Clb3d'}[\mathrm{Ama1}] + k_{Clb3d''}[\mathrm{Ama1_T}] + k_{Clb3dCdc20}[\mathrm{APC\text{-}P{:}Cdc20}]\right)[\mathrm{Clb3}] \\ &+ \alpha_{Clb3}\, k_{Clb3s'}(1-f(t))e^{-(t-T)/\tau_{mRNA}}, \quad (5)\end{aligned}$$

$$\frac{d[\mathrm{Ndt80}]}{dt} = k_{Ndt80s} + k_{Ndt80s'}\frac{[\mathrm{Ndt80}]^{\mathrm{n}}}{K^{n}_{Ndt80} + [\mathrm{Ndt80}]^{\mathrm{n}}} - \left(k_{Ndt80d} + k_{Ndt80d'}[\mathrm{Ama1}]\right)[\mathrm{Ndt80}], \quad (6)$$

$$\frac{d[\mathrm{Cdc20_T}]}{dt} = k_{Cdc20s} + k_{Cdc20s'}[\mathrm{Ndt80}] - k_{Cdc20d}[\mathrm{Cdc20_T}] - k_{Cdc20d'}[\mathrm{APC\text{-}P{:}Cdc20}], \quad (7)$$

$$\begin{aligned}\frac{d[\mathrm{APC\text{-}P}]}{dt} &= k_{APCClb1p}[\mathrm{Clb1}]\frac{[\mathrm{APC}]}{J_{APCClb1} + [\mathrm{APC}]} + k_{APCClb3p}[\mathrm{Clb3}]\frac{[\mathrm{APC}]}{J_{APCClb3} + [\mathrm{APC}]} \\ &+ k_{APCClb4p}[\mathrm{Clb4}]\frac{[\mathrm{APC}]}{J_{APCClb4} + [\mathrm{APC}]} - k_{APCdp}\frac{[\mathrm{APC\text{-}P}]}{J_{APC-P} + [\mathrm{APC\text{-}P}]}, \quad (8)\end{aligned}$$

$$\begin{aligned}\frac{d[\mathrm{APC\text{-}P{:}Cdc20}]}{dt} &= k_{APC-P:Cdc20a}[\mathrm{APC\text{-}P}]\frac{[\mathrm{Cdc20_T}] - [\mathrm{APC\text{-}P{:}Cdc20}]}{J_{APC-P:Cdc20a} + [\mathrm{Cdc20_T}] - [\mathrm{APC\text{-}P{:}Cdc20}]} \\ &- k_{APC-P:Cdc20d}\frac{[\mathrm{APC\text{-}P{:}Cdc20}]}{J_{APC-P:Cdc20d} + [\mathrm{APC\text{-}P{:}Cdc20}]}, \quad (9)\end{aligned}$$

$$\begin{aligned}\frac{d[\mathrm{Cdc5_A}]}{dt} &= \left(k_{Cdc5a} + k_{Cdc5a'}[\mathrm{Clb1}] + k_{Cdc5a''}[\mathrm{Clb4}] + k_{Cdc5a'''}[\mathrm{Clb3}]\right)\left([\mathrm{Cdc5_T}] - [\mathrm{Cdc5_A}]\right) \\ &- k_{Cdc5i}[\mathrm{Cdc5_A}] - \left(k_{Cdc5d} + k_{Cdc5d'}[\mathrm{Ama1}] + k_{Cdc5d''}[\mathrm{Ama1_T}]\right)[\mathrm{Cdc5_A}], \quad (10)\end{aligned}$$

$$\frac{d[\mathrm{Cdc5_T}]}{dt} = k_{Cdc5s} + k_{Cdc5s'}[\mathrm{Ndt80}] - \left(k_{Cdc5d} + k_{Cdc5d'}[\mathrm{Ama1}] + k_{Cdc5d''}[\mathrm{Ama1_T}]\right)[\mathrm{Cdc5_T}], \quad (11)$$

$$\frac{d[\text{Ama1-P}]}{dt} = (k_{Ama1i} + k_{Ama1i'}[\text{Clb1}] + k_{Ama1i'}[\text{Clb4}] + k_{Ama1i'}[\text{Clb3}]) \frac{[\text{Ama1}]}{J_{Ama1} + [\text{Ama1}]} - k_{Ama1a}\frac{[\text{Ama1-P}]}{J_{Ama1'} + [\text{Ama1-P}]} - k_{Ama1d'}[\text{Ama1-P}], \tag{12}$$

$$\frac{d[\text{Ama1}]}{dt} = k_{Ama1s} - k_{Ama1d}[\text{Ama1}] + \alpha_{Ama1}\, k_{Ama1s}(1 - f(t))e^{-(t-T)/\tau_{mRNA}} - (k_{Ama1i} + k_{Ama1i'}[\text{Clb1}] + k_{Ama1i'}[\text{Clb3}] + k_{Ama1i'}[\text{Clb4}]) \frac{[\text{Ama1}]}{J_{Ama1} + [\text{Ama1}]} + k_{Ama1a}\frac{[\text{Ama1-P}]}{J_{Ama1'} + [\text{Ama1-P}]}, \tag{13}$$

Additionally,

$$[\text{APC}] = [\text{APC}_\text{T}] - [\text{APC-P}] - [\text{APC-P:Cdc20}]. \tag{14}$$

1.4 Model Parameters, Initial Conditions

Table S1: Clb1 Parameters

Parameter	Value
k_{Clb1s}	0.04
$k_{Clb1s'}$	0.024
k_{Clb1d}	0.1
$k_{Clb1d'}$	0.5
$k_{Clb1d''}$	0.02
$k_{Clb1dCdc20}$	0.8

Table S2: APC/C$^{\text{Cdc20}}$ Parameters

Parameter	Value
$k_{APC-P:Cdc20a}$	0.25
$k_{APC-P:Cdc20d}$	0.32
$J_{APC-P:Cdc20a}$	0.1
$J_{APC-P:Cdc20d}$	0.1

Table S3: Clb3 Parameters

Parameter	Value
k_{Clb3s}	0.002
$k_{Clb3s'}$	0.000114
k_{Clb3d}	0.34
$k_{Clb3d'}$	0.2
$k_{Clb3d''}$	0.02
$k_{Clb3dCdc20}$	0.4
$\alpha_{Clb3}k_{Clb3s'}$	0.4
τ_{mRNA}	25

Table S4: Cdc5 Parameters

Parameter	Value
k_{Cdc5a}	0.1
k_{Cdc5i}	2
k_{Cdc5d}	0.04
$k_{Cdc5d'}$	0.003
$k_{Cdc5d''}$	0.002
$k_{Cdc5a'}$	1.2
$k_{Cdc5a''}$	1.2
$k_{Cdc5a'''}$	2
k_{Cdc5s}	0.004
$k_{Cdc5s'}$	0.027

Table S5: Clb4 Parameters

Parameter	Value
k_{Clb4s}	0.0008
$k_{Clb4s'}$	0.009
k_{Clb4d}	0.02
$k_{Clb4d'}$	0.2
$k_{Clb4d''}$	0.02
$k_{Clb4dCdc20}$	0.2

Table S6: Ama1 Parameters

Parameter	Value
k_{Ama1i}	0.005
$k_{Ama1i'}$	0.5
J_{Ama1}	0.1
k_{Ama1a}	0.1
$J_{Ama1'}$	0.1
k_{Ama1s}	0.04
k_{Ama1d}	0.02
$k_{Ama1d'}$	0.02
$\alpha_{Ama1}k_{Ama1s}$	0.4
τ_{mRNA}	25

Table S7: Ndt80 Parameters

Parameter	Value
k_{Ndt80s}	0.01
$k_{Ndt80s'}$	2
k_{Ndt80d}	0.093
$k_{Ndt80d'}$	0.013
(n, K_{Ndt80})	$(1.6, 10.5)$

Table S8: Cdc20 Parameters

Parameter	Value
k_{Cdc20s}	0.1
$k_{Cdc20s'}$	0.001
k_{Cdc20d}	0.05
$k_{Cdc20d'}$	0.02

Table S9: APC/C Parameters

$k_{APCClb1p}$	0.09
$k_{APCClb4p}$	0.09
$k_{APCClb3p}$	0.09
$J_{APCClb1}$	0.001
$J_{APCClb4}$	0.001
$J_{APCClb3}$	0.001
k_{APCdp}	0.072
J_{APC-P}	0.01
$[\mathrm{APC_T}]$	5

Table S10: Initial Conditions

Clb1	1.125
Clb4	0.12
Clb3	0
Ndt80	5
$\mathrm{Cdc20_T}$	2
APC-P	0
APC-P:Cdc20	0.1
$\mathrm{Cdc5_A}$	0.25
$\mathrm{Cdc5_T}$	0.75
Ama1-P	0
Ama1	0

1.5 Supplementary Figures

For completeness, Figures S1-S7 show the numerical solutions to all variables of the exit network for wild-type and various mutants, given by Eqs. (3)-(14), using initial conditions given in Table S10 for wild-type. For each deletion, if the initial concentration is nonzero, it is set equal to zero. Concentrations are in arbitrary units (a.u.). The horizontal axis denotes time since the start of meiosis II. In the main manuscript, only a subset of the solutions are shown for greater clarity. Figure S8 shows the mRNA-Rim4 dissociation dynamics for different timings of mRNA release, and Figure S9 shows the resulting total Cdk1-Clb concentration and threshold crossing. Figure S10 shows results for a single, total Cdk-Clb species.

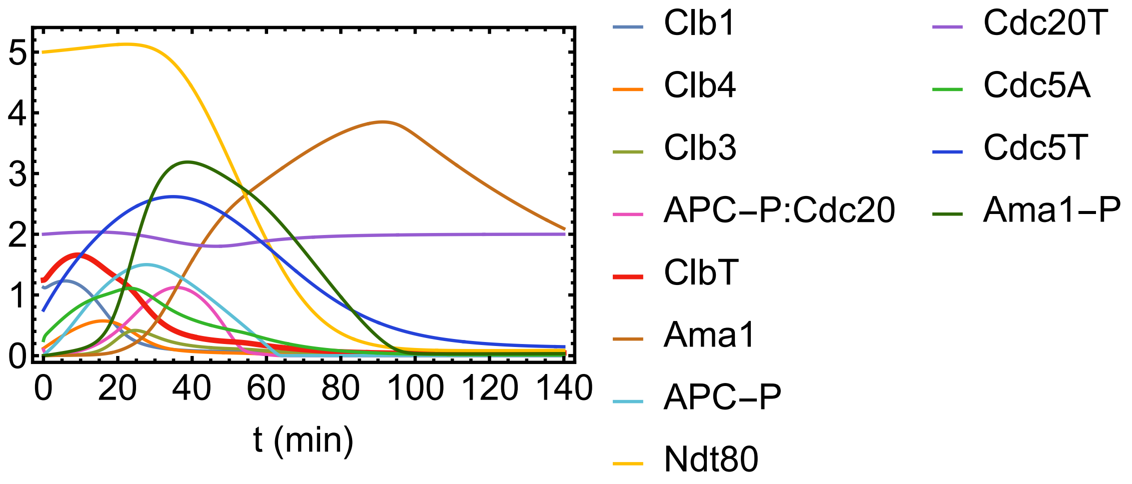

Figure S1: Wild-type full model dynamics (Fig. 3B)

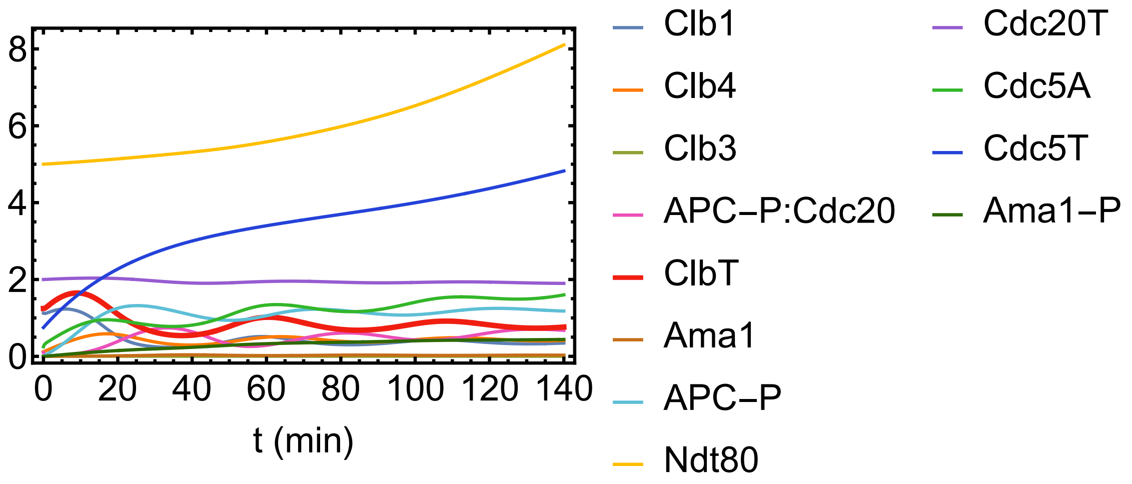

Figure S2: No Rim4 degradation ($f(t;T,\tau_p)=1$), full model dynamics (Fig. 3C)

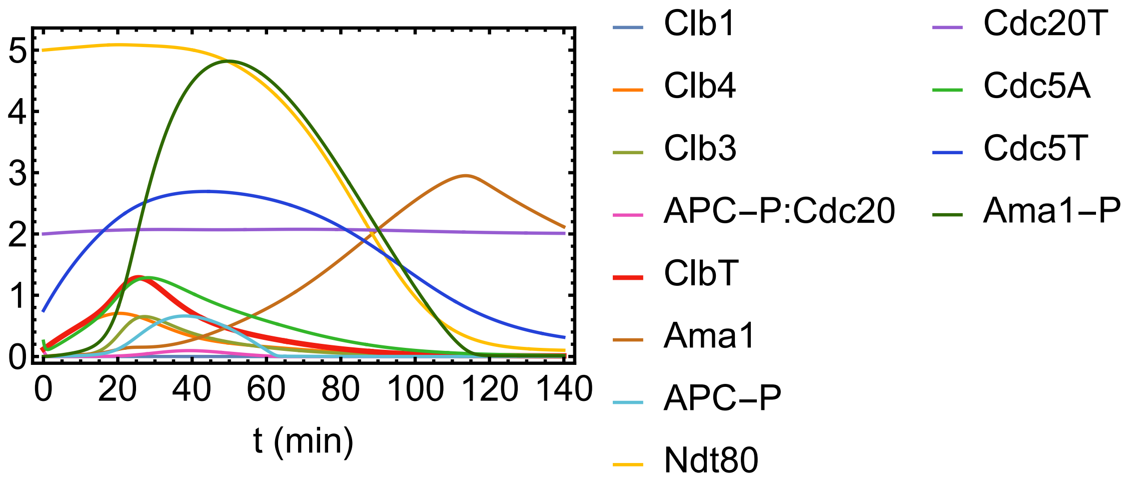

Figure S3: Clb1 deletion full model dynamics (Fig. 3D)

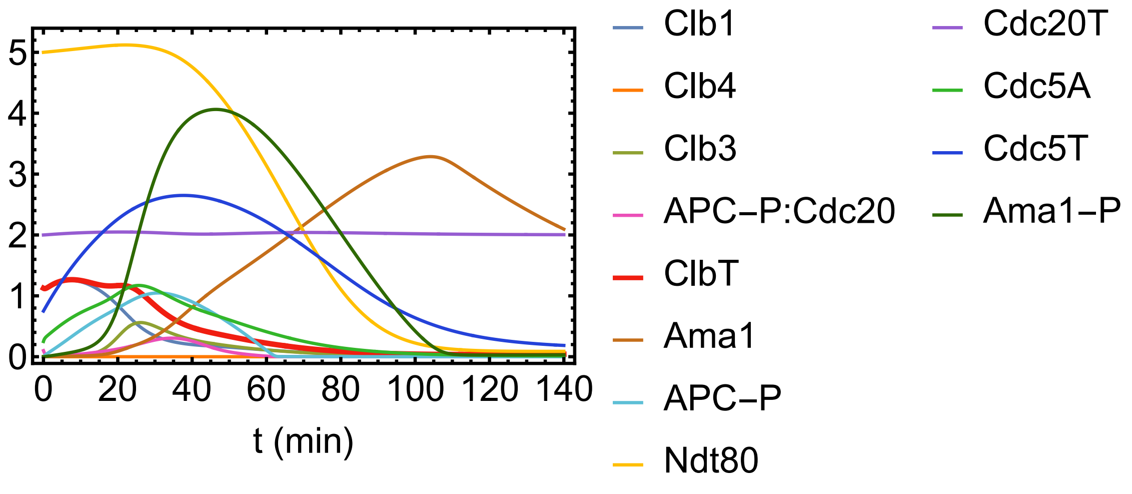

Figure S4: Clb4 deletion full model dynamics (Fig. 3E)

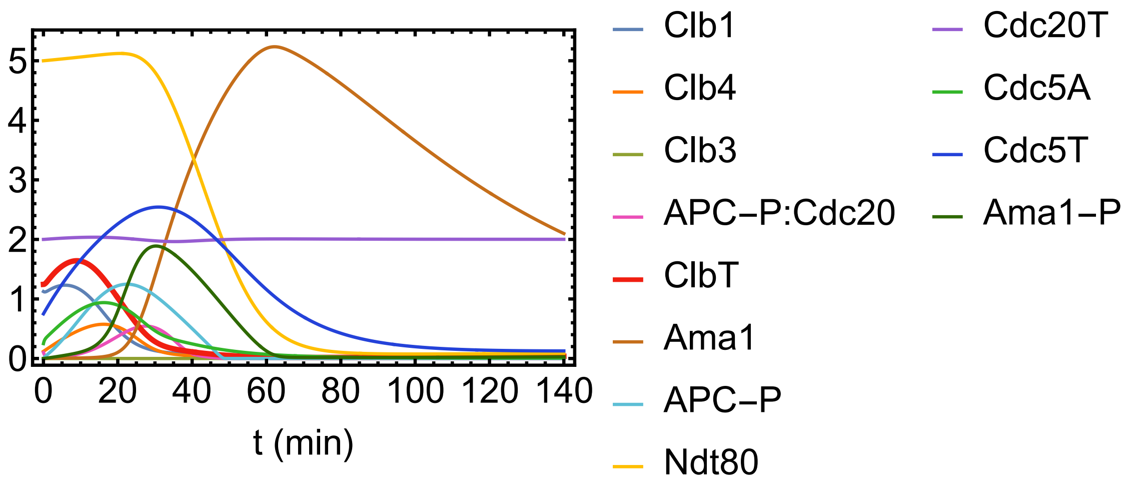

Figure S5: Clb3 deletion full model dynamics (Fig. 3F)

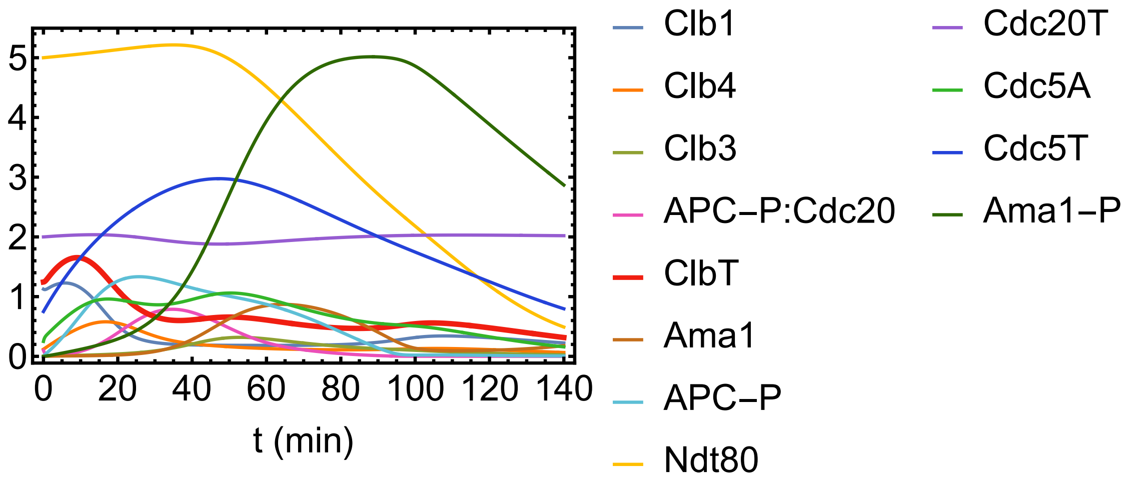

Figure S6: *rim4-47A-2* full model dynamics (Fig. 4B)

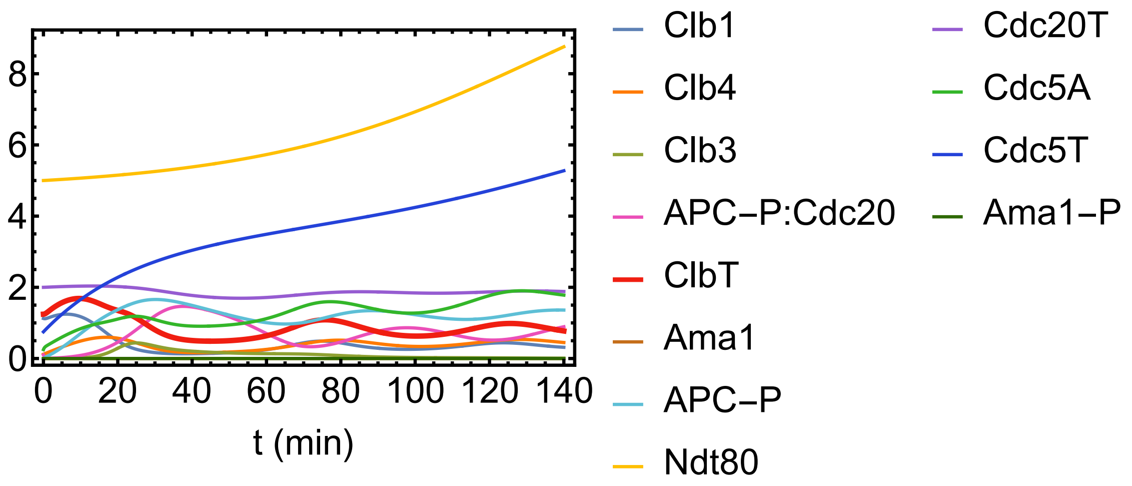

Figure S7: Ama1 deletion full model dynamics (Fig. 5A)

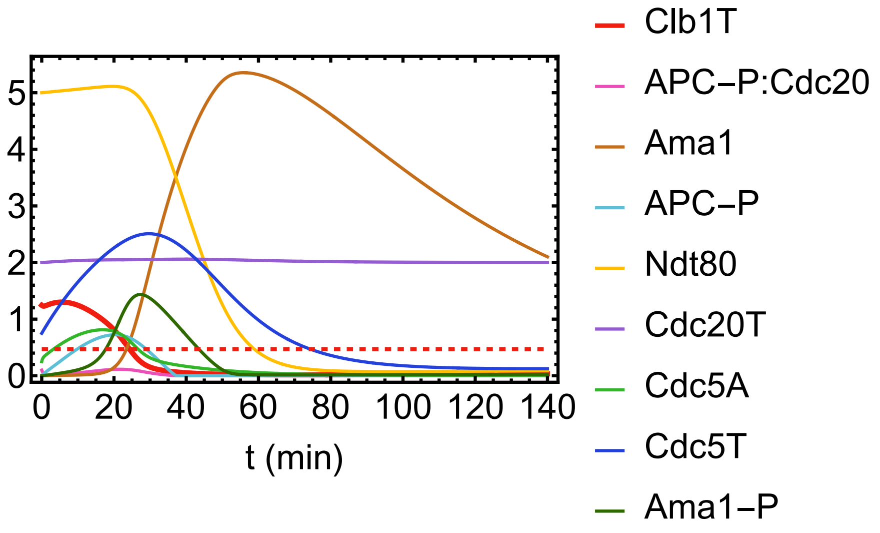

Figure S8: Single ClbT species. Clb1, Clb4, and Clb3 are replaced by a single, total Clb variable in the model, ClbT, with kinetic parameters given by that of Clb1. We note that the total Clb concentration drops below threshold at $t_{\text{threshold}} = 24.3$ min, corresponding to a faster exit from meiosis II (compared to the experimentally observed 38 ± 18 min). Since the values of kinetic parameters for Clb1 and Clb4 are comparable, the dynamics shown here is similar to the Clb3 mutant (Fig. S5). The later expression of Clb3 upon Rim4 release, even at modest concentrations relative to Clb1 and Clb4, serves to maintain higher levels of total Cdk1-Clb kinase activity for longer, thereby prolonging exit.

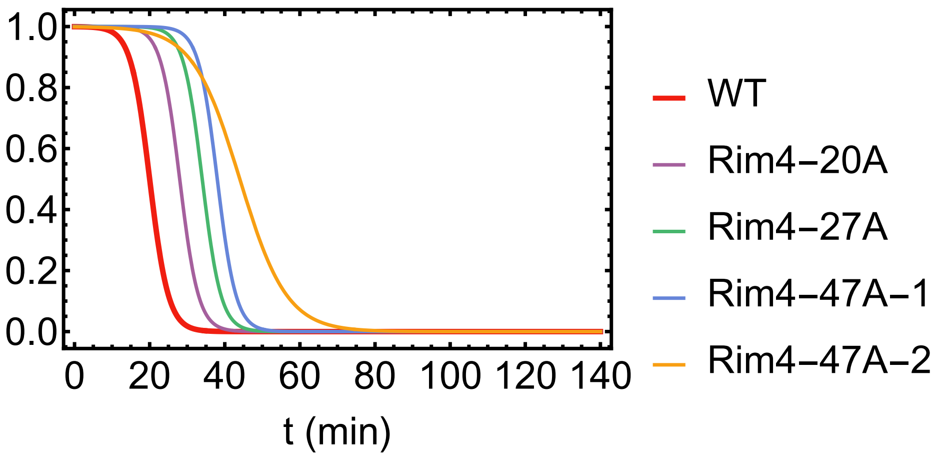

Figure S9: mRNA-Rim4 dissociation and Rim4 clearance given by Eq. 1. Red: $T = 20\,\text{min}, \tau_p^{-1} = 0.2\ \text{min}^{-1}$ (WT). Purple: $T = 28\,\text{min}, \tau_p^{-1} = 0.2\ \text{min}^{-1}$ (*rim4-20A*). Green: $T = 34\,\text{min}, \tau_p^{-1} = 0.2\ \text{min}^{-1}$ (*rim4-27A*). Blue: $T = 38\,\text{min}, \tau_p^{-1} = 0.2\ \text{min}^{-1}$ (*rim4-47A-1*). Yellow: $T = 44\,\text{min}, \tau_p^{-1} = 0.08\ \text{min}^{-1}$ (*rim4-47A-2*). To describe Rim4 phosphorylation mutants, the onset time, T, and rate of mRNA release (and Rim4 clearance), plausibly change with respect to wild-type. The horizontal axis is time in meiosis II, and on the vertical axis, a value of 1.0 corresponds to complete sequestration of mRNA by Rim4, and 0.0 corresponds to complete release of mRNA and Rim4 clearance.

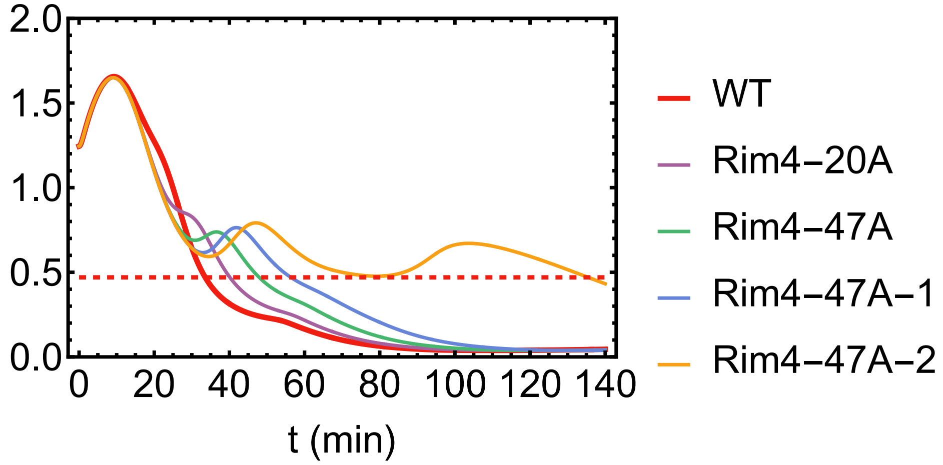

Figure S10: Total Cdk1-Clb concentration for WT and different Rim4 mutants. Red: WT ($T = 20\,\text{min}, \tau_p^{-1} = 0.2\ \text{min}^{-1}$). Purple: *rim4-20A* ($T = 28\,\text{min}, \tau_p^{-1} = 0.2\ \text{min}^{-1}$). Green: *rim4-27A* ($T = 34\,\text{min}, \tau_p^{-1} = 0.2\ \text{min}^{-1}$). Blue: *rim4-47A-1* ($T = 38\,\text{min}, \tau_p^{-1} = 0.2\ \text{min}^{-1}$). Orange: *rim4-47A-2* ($T = 44\,\text{min}, \tau_p^{-1} = 0.08\ \text{min}^{-1}$). Dashed red: Total Cdk1-Clb threshold. We note that with increasing T, and therefore delay in dissociation of mRNA from Rim4 (and Rim4 clearance), threshold crossing is delayed, until oscillatory dynamics sets in.

References

[1] Mansooreh Ahmadian, John J. Tyson, Jean Peccoud, and Yang Cao. A hybrid stochastic model of the budding yeast cell cycle. *npj Systems Biology and Applications*, 6(1), March 2020.

[2] Uri Alon. *An introduction to systems biology: design principles of biological circuits.* Chapman and Hall/CRC, 2019.

[3] Debashis Barik, David A. Ball, Jean Peccoud, and John J. Tyson. A Stochastic Model of the Yeast Cell Cycle Reveals Roles for Feedback Regulation in Limiting Cellular Variability. *PLoS Computational Biology*, 12(12):e1005230, December 2016.

[4] Debashis Barik, William T Baumann, Mark R Paul, Bela Novak, and John J Tyson. A model of yeast cell-cycle regulation based on multisite phosphorylation. *Molecular Systems Biology*, 6(1), January 2010.

[5] Dorjsuren Battogtokh and John J. Tyson. Bifurcation analysis of a model of the budding yeast cell cycle. *Chaos: An Interdisciplinary Journal of Nonlinear Science*, 14(3):653–661, September 2004.

[6] Tanvi Bhola, Orsolya Kapuy, and P. K. Vinod. Computational modelling of meiotic entry and commitment. *Scientific Reports*, 8(1), January 2018.

[7] William Bialek and Sima Setayeshgar. Physical limits to biochemical signaling. *Proceedings of the National Academy of Sciences*, 102(29):10040–10045, 2005.

[8] William Bialek and Sima Setayeshgar. Cooperativity, sensitivity, and noise in biochemical signaling. *Physical Review Letters*, 100(25):258101, 2008.

[9] Steven Boeynaems, Simon Alberti, Nicolas L. Fawzi, Tanja Mittag, Magdalini Polymenidou, Frederic Rousseau, Joost Schymkowitz, James Shorter, Benjamin Wolozin, Ludo Van Den Bosch, Peter Tompa, and Monika Fuxreiter. Protein Phase Separation: A New Phase in Cell Biology. *Trends in Cell Biology*, 28(6):420–435, June 2018.

[10] Clifford P Brangwynne, Peter Tompa, and Rohit V Pappu. Polymer physics of intracellular phase transitions. *Nature Physics*, 11(11):899–904, 2015.

[11] Gloria A Brar, Moran Yassour, Nir Friedman, Aviv Regev, Nicholas T Ingolia, and Jonathan S Weissman. High-resolution view of the yeast meiotic program revealed by ribosome profiling. *Science*, 335(6068):552–557, 2012.

[12] Kevin S. Brown and James P. Sethna. Statistical mechanical approaches to models with many poorly known parameters. *Physical Review E*, 68(2):021904, 2003.

[13] Katherine C Chen, Laurence Calzone, Attila Csikasz-Nagy, Frederick R Cross, Bela Novak, and John J Tyson. Integrative analysis of cell cycle control in budding yeast. *Molecular Biology of the Cell*, 15(8):3841–3862, 2004.

[14] Katherine C. Chen, Attila Csikasz-Nagy, Bela Gyorffy, John Val, Bela Novak, and John J. Tyson. Kinetic Analysis of a Molecular Model of the Budding Yeast Cell Cycle. *Molecular Biology of the Cell*, 11(1):369–391, January 2000.

[15] Andrea Ciliberto, Anna Lukács, Attila Tóth, John J. Tyson, and Bela Novak. Rewiring the Exit from Mitosis. *Cell Cycle*, 4(8):4107–4112, August 2005.

[16] Katrina F Cooper and Randy Strich. Meiotic control of the APC/C: similarities & differences from mitosis. *Cell Division*, 6(1):16, 2011.

[17] Attila Csikász-Nagy, Dorjsuren Battogtokh, Katherine C Chen, Béla Novák, and John J Tyson. Analysis of a generic model of eukaryotic cell-cycle regulation. *Biophysical journal*, 90(12):4361–4379, 2006.

[18] Michael B Elowitz, Arnold J Levine, Eric D Siggia, and Peter S Swain. Stochastic gene expression in a single cell. *Science*, 297(5584):1183–1186, 2002.

[19] James E. Ferrell and Sang Hoon Ha. Ultrasensitivity part I: Michaelian responses and zero-order ultrasensitivity. *Trends in Biochemical Sciences*, 39(10):496–503, October 2014.

[20] James E. Ferrell and Sang Hoon Ha. Ultrasensitivity part III: cascades, bistable switches, and oscillators. *Trends in Biochemical Sciences*, 39(12):612–618, December 2014.

[21] James E. Ferrell, Jr, and Sang Hoon Ha. Ultrasensitivity part II: multisite phosphorylation, stoichiometric inhibitors, and positive feedback. *Trends in Biochemical Sciences*, 39(11):556–569, November 2014.

[22] Joseph V Geisberg, Zarmik Moqtaderi, Xiaochun Fan, Fatih Ozsolak, and Kevin Struhl. Global analysis of mrna isoform half-lives reveals stabilizing and destabilizing elements in yeast. *Cell*, 156(4):812–824, 2014.

[23] Sina Ghaemmaghami, Won-Ki Huh, Kiowa Bower, Russell W Howson, Archana Belle, Noah Dephoure, Erin K O'Shea, and Jonathan S Weissman. Global analysis of protein expression in yeast. *Nature*, 425(6959):737–741, 2003.

[24] Daniel T Gillespie. Exact stochastic simulation of coupled chemical reactions. *The Journal of Physical Chemistry*, 81(25):2340–2361, 1977.

[25] Daniel T Gillespie. Stochastic simulation of chemical kinetics. *Annu. Rev. Phys. Chem.*, 58(1):35–55, 2007.

[26] A Goldbeter and D E Koshland. An amplified sensitivity arising from covalent modification in biological systems. *Proceedings of the National Academy of Sciences*, 78(11):6840–6844, November 1981.

[27] Albert Goldbeter. A minimal cascade model for the mitotic oscillator involving cyclin and cdc2 kinase. *Proceedings of the National Academy of Sciences*, 88(20):9107–9111, 1991.

[28] Ryan N Gutenkunst, Joshua J Waterfall, Fergal P Casey, Kevin S Brown, Christopher R Myers, and James P Sethna. Universally sloppy parameter sensitivities in systems biology models. *PLoS Computational Biology*, 3(10):e189, 2007.

[29] S Grace Herod, Annie Dyatel, Stefanie Hodapp, Marko Jovanovic, and Luke E Berchowitz. Clearance of an amyloid-like translational repressor is governed by 14-3-3 proteins. *Cell Reports*, 39(5), 2022.

[30] Adam Klosin, Florian Oltsch, Tyler Harmon, Alf Honigmann, Frank Jülicher, Anthony A Hyman, and Christoph Zechner. Phase separation provides a mechanism to reduce noise in cells. *Science*, 367(6476):464–468, 2020.

[31] Daniel E Koshland Jr, George Némethy, and David Filmer. Comparison of experimental binding data and theoretical models in proteins containing subunits. *Biochemistry*, 5(1):365–385, 1966.

[32] Helene Labit, Kazuyuki Fujimitsu, N Sumru Bayin, Tohru Takaki, Julian Gannon, and Hiroyuki Yamano. Dephosphorylation of Cdc20 is required for its C-box-dependent activation of the APC/C: Dephosphorylation-driven activation of the APC/C. *The EMBO Journal*, 31(15):3351–3362, August 2012.

[33] Anne M. MacKenzie and Soni Lacefield. Cdk regulation of meiosis: Lessons from saccharomyces cerevisiae and schizosaccharomyces pombe. *Genes*, 11(7):723, 2020.

[34] Sarah Marzen, Hernan G Garcia, and Rob Phillips. Statistical mechanics of monod–wyman–changeux (mwc) models. *Journal of molecular biology*, 425(9):1433–1460, 2013.

[35] Ron Milo, Shai Shen-Orr, Shalev Itzkovitz, Nadav Kashtan, Dmitri Chklovskii, and Uri Alon. Network motifs: simple building blocks of complex networks. *Science*, 298(5594):824–827, 2002.

[36] Jacques Monod, Jeffries Wyman, and Jean-Pierre Changeux. On the nature of allosteric transitions: a plausible model. *Journal of Molecular Biology*, 12(1):88–118, 1965.

[37] John RS Newman, Sina Ghaemmaghami, Jan Ihmels, David K Breslow, Matthew Noble, Joseph L DeRisi, and Jonathan S Weissman. Single-cell proteomic analysis of s. cerevisiae reveals the architecture of biological noise. *Nature*, 441(7095):840–846, 2006.

[38] Bela Novak and John J Tyson. Modeling the control of dna replication in fission yeast. *Proceedings of the National Academy of Sciences*, 94(17):9147–9152, 1997.

[39] Béla Novák and John J. Tyson. Design principles of biochemical oscillators. *Nature Reviews Molecular Cell Biology*, 9(12):981–991, December 2008.

[40] Elwy Okaz, Orlando Argüello-Miranda, Aliona Bogdanova, PK Vinod, Jesse J Lipp, Zuzana Markova, Ievgeniia Zagoriy, Bela Novak, and Wolfgang Zachariae. Meiotic prophase requires proteolysis of m phase regulators mediated by the meiosis-specific apc/cama1. *Cell*, 151(3):603–618, 2012.

[41] Theodore J Perkins and Peter S Swain. Strategies for cellular decision-making. *Molecular systems biology*, 5(1):326, 2009.

[42] Hong Qian. Thermodynamic and Kinetic Analysis of Sensitivity Amplification in Biological Signal Transduction. *Biophysical Chemistry*, 105(2-3):585–593, September 2003. arXiv:physics/0207049.

[43] Renping Qiao, Florian Weissmann, Masaya Yamaguchi, Nicholas G Brown, Ryan VanderLinden, Richard Imre, Marc A Jarvis, Michael R Brunner, Iain F Davidson, Gabriele Litos, et al. Mechanism of apc/c^{cdc20} activation by mitotic phosphorylation. *Proceedings of the National Academy of Sciences*, 113(19):E2570–E2578, 2016.

[44] Arpan Kumar Rai, Jia-Xuan Chen, Matthias Selbach, and Lucas Pelkmans. Kinase-controlled phase transition of membraneless organelles in mitosis. *Nature*, 559(7713):211–216, July 2018.

[45] Arjun Raj and Alexander Van Oudenaarden. Nature, nurture, or chance: stochastic gene expression and its consequences. *Cell*, 135(2):216–226, 2008.

[46] Carlos Salazar and Thomas Höfer. Multisite protein phosphorylation – from molecular mechanisms to kinetic models. *The FEBS Journal*, 276(12):3177–3198, June 2009.

[47] Y Shin and CP Brangwynne. Liquid phase condensation in cell physiology and disease. science 357, eaaf4382, 2017.

[48] Thomas Stoeger, Nico Battich, and Lucas Pelkmans. Passive Noise Filtering by Cellular Compartmentalization. *Cell*, 164(6):1151–1161, March 2016.

[49] Peter S Swain, Michael B Elowitz, and Eric D Siggia. Intrinsic and extrinsic contributions to stochasticity in gene expression. *Proceedings of the National Academy of Sciences*, 99(20):12795–12800, 2002.

[50] Mukund Thattai and Alexander Van Oudenaarden. Intrinsic noise in gene regulatory networks. *Proceedings of the National Academy of Sciences*, 98(15):8614–8619, 2001.

[51] Mark K. Transtrum, Benjamin B. Machta, Kevin S. Brown, Bryan C. Daniels, Christopher R. Myers, and James P. Sethna. Perspective: Sloppiness and emergent theories in physics, biology, and beyond. *The Journal of Chemical Physics*, 143(1):010901, 2015.

[52] Mark K. Transtrum, Benjamin B. Machta, and James P. Sethna. Why are nonlinear fits to data so challenging? *Physical Review Letters*, 104(6):060201, 2010.

[53] Daisuke Tsuchiya, Ying Yang, and Sara Lacefield. Positive feedback of ndt80 expression ensures irreversible meiotic commitment in budding yeast. *PLoS Genetics*, 10(6):e1004398, 2014.

[54] John J Tyson and Bela Novak. Regulation of the Eukaryotic Cell Cycle: Molecular Antagonism, Hysteresis, and Irreversible Transitions. *Journal of Theoretical Biology*, 210(2):249–263, May 2001.

[55] John J. Tyson and Béla Novák. Time-keeping and decision-making in the cell cycle. *Interface Focus*, 12(4), August 2022.

[56] Vladimir N Uversky, Irina M Kuznetsova, Konstantin K Turoverov, and Boris Zaslavsky. Intrinsically disordered proteins as crucial constituents of cellular aqueous two phase systems and coacervates. *FEBS Letters*, 589(1):15–22, 2015.

[57] Fei Wang, Rudian Zhang, Wenzhi Feng, Dai Tsuchiya, Olivia Ballew, Jiajia Li, Vladimir Denic, and Soni Lacefield. Autophagy of an amyloid-like translational repressor regulates meiotic exit. *Developmental Cell*, 52(2):141–151, 2020.

[58] Jennifer T Wang, Jarrett Smith, Bi-Chang Chen, Helen Schmidt, Dominique Rasoloson, Alexandre Paix, Bramwell G Lambrus, Deepika Calidas, Eric Betzig, and Geraldine Seydoux. Regulation of rna granule dynamics by phosphorylation of serine-rich, intrinsically disordered proteins in c. elegans. *Elife*, 3:e04591, 2014.

[59] Jin Wang, Chunhe Li, and Erkang Wang. Potential and flux landscapes quantify the stability and robustness of budding yeast cell cycle network. *Proceedings of the National Academy of Sciences*, 107(18):8195–8200, May 2010.

[60] Joshua J. Waterfall, Fergal P. Casey, Joshua N. Gutenkunst, Kevin S. Brown, Christopher R. Myers, Piet W. Brouwer, and James P. Sethna. Sloppy-model universality class and the vandermonde matrix. *Physical Review Letters*, 97(15):150601, 2006.

[61] Christoph A Weber, David Zwicker, Frank Jülicher, and Chiu Fan Lee. Physics of active emulsions. *Reports on Progress in Physics*, 82(6):064601, June 2019.

[62] Rachel M Welles, Kandarp A Sojitra, Mikael V Garabedian, Boao Xia, Wentao Wang, Muyang Guan, Roshan M Regy, Elizabeth R Gallagher, Daniel A Hammer, Jeetain Mittal, et al. Determinants that enable disordered protein assembly into discrete condensed phases. *Nature chemistry*, 16(7):1062–1072, 2024.

[63] Edward Winter. The sum1/ndt80 transcriptional switch and commitment to meiosis in saccharomyces cerevisiae, 2012.

[64] Christoph Zechner and Frank Jülicher. Concentration buffering and noise reduction in non-equilibrium phase-separating systems. *Cell Systems*, 16(2), 2025.

[65] Huaiying Zhang, Shana Elbaum-Garfinkle, Erin M. Langdon, Nicole Taylor, Patricia Occhipinti, Andrew A. Bridges, Clifford P. Brangwynne, and Amy S. Gladfelter. RNA Controls PolyQ Protein Phase Transitions. *Molecular Cell*, 60(2):220–230, October 2015.

Table S11: Yeast strain list

Strain number	Genotype
LY4472	*MAT***a***/α*, *ZIP1-GFP-700/+*, *TUB1pr-GFP-TUB1*:URA3/*TUB1pGFP*-TUB1:URA3, *SPC42-mCherry*:*hphMX*/+, *ama1::kanMX/ama1::kanMX*
LY8899	*MAT***a***/α*, *HIS3pr-yomRuby2-TUB1:URA3/HIS3pr-yomRuby2-TUB1:URA3, SPC42-GFP:HIS3/+*
LY9468	*MAT***a***/α*, *HIS3pr-yomRuby2-TUB1:URA3/HIS3pr-yomRuby2-TUB1:URA3, CDC14-GFP:HIS3/CDC14-GFP:HIS3*
LY9528	*MAT***a***/α*, *HIS3pr-yomRuby2-TUB1:URA3/HIS3pr-yomRuby2-TUB1:URA3*, *CDC14-GFP:HIS3/CDC14-GFP:HIS3*, *clb1::kanMX/clb1::kanMX*
LY9529	*MAT***a***/α*, *HIS3pr-yomRuby2-TUB1:URA3/HIS3pr-yomRuby2-TUB1:URA3*, *CDC14-GFP:HIS3/CDC14-GFP:HIS3*, *clb3::natMX/clb3::natMX*
LY9530	*MAT***a***/α*, *HIS3pr-yomRuby2-TUB1:URA3/HIS3pr-yomRuby2-TUB1:URA3*, *CDC14-GFP:HIS3/CDC14-GFP:HIS3*, *clb4::hphMX/clb4::hphMX*
LY9798	*MAT***a***/α*, *HIS3pr-yomRuby2-TUB1:URA3/HIS3pr-yomRuby2-TUB1:URA3*, *CDC14-GFP:kanMX/CDC14-GFP:kanMX, rim4-20A-3V5:HIS3/rim4-20A-3V5:HIS3*
LY9797	*MAT***a***/α*, *HIS3pr-yomRuby2-TUB1:URA3/HIS3pr-yomRuby2-TUB1:URA3*, *CDC14-GFP:kanMX/CDC14-GFP:kanMX*, *rim4-27A-3V5:HIS3/rim4-27A-3V5:HIS3*
LY9803	*MAT***a***/α*, *HIS3pr-yomRuby2-TUB1:URA3/HIS3pr-yomRuby2-TUB1:URA3*, *CDC14-GFP:HIS3/CDC14-GFP:HIS3*, *rim4-47A-3V5:kanMX/rim4-47A-3V5:kanMX*
LY9809	*MAT***a***/α*, *HIS3pr-yomRuby2-TUB1:URA3/HIS3pr-yomRuby2-TUB1:URA3*, *CDC14-GFP:HIS3/CDC14-GFP:HIS3*, *clb1::kanMX/clb1::kanMX, clb4::hphMX/clb4::hphMX*
LY9810	*MAT***a***/α*, *HIS3pr-yomRuby2-TUB1:URA3/HIS3pr-yomRuby2-TUB1:URA3*, *CDC14-GFP:HIS3/CDC14-GFP:HIS3*, *clb3*::*natMX/clb3::natMX, clb4::hphMX/clb4::hphMX*
LY9861	*MAT***a***/α*, *HIS3pr-yomRuby2-TUB1:URA3/HIS3pr-yomRuby2-TUB1:URA3*, *CDC14-GFP:HIS3/CDC14-GFP:HIS3*, *clb1*::*kanMX/clb1::kanMX, clb3::natMX/clb3::natMX*
LY9933	*MAT***a***/α*, *HIS3pr-yomRuby2-TUB1:URA3/HIS3pr-yomRuby2-TUB1:URA3*, *CDC14-GFP:HIS3/CDC14-GFP:HIS3*, *ama1::kanMX/ama1::kanMX*
LY10537	*MAT***a***/α*, *HIS3pr-yomRuby2-TUB1:URA3/HIS3pr-yomRuby2-TUB1:URA3*, *SPC42-GFP:HIS3/+, rim4-47A-3V5:kanMX*/*rim4-47A*-3V5:kanMX
LY10732	*MAT***a***/α*, *HIS3pr-yomRuby2-TUB1:URA3/HIS3pr-yomRuby2-TUB1:URA3*, *SPC42-GFP:HIS3/+*, *rim4-27A-3V5:HIS3/rim4-27A-3V5:HIS3*
LY10735	*MAT***a***/α*, *HIS3pr-yomRuby2-TUB1:URA3/HIS3pr-yomRuby2-TUB1:URA3*, *SPC42-GFP:HIS3/+*, *rim4-20A-3V5:HIS3/rim4-20A-3V5:HIS3*

SUPPLEMENTARY INFORMATION

Supplementary Methods
Description of Mathematical Model for Meiotic Exit
References
Model Equations

Supplementary Tables
Table S1. Clb1 parameters.
Table S2. APC/C^{Cdc20} parameters.
Table S3. Clb3 parameters.
Table S4. Cdc5 parameters.
Table S5. Clb4 parameters.
Table S6. Ama1 parameters.
Table S7. Ndt80 parameters.
Table S8. Cdc20 parameters.
Table S9. APC/C parameters.
Table S10. Initial conditions.
Table S11. Yeast Strains used in this study.

Supplementary Figures

Figures S1-S7 show the numerical solutions to all variables of the exit network, given by equations 3-14. In the main figure, only a subset of variables is shown for greater clarity.

Figure S1 (related to Figure 3B). Dynamics for all exit network elements in wildtype cells. Concentrations are in arbitrary units (a.u.) on the vertical axes. The horizontal axes denote time since the start of meiosis II.

Figure S2 (related to Figure 3C). Dynamics for all exit network elements in cells with no Rim4 degradation. Concentrations are in arbitrary units (a.u.) on the vertical axis. The horizontal axis denotes time since the start of meiosis II.

Figure S3 (related to Figure 3D). Dynamics for all exit network elements in *clb1Δ* cells, with the concentration of Clb1 set to zero. Concentrations are in arbitrary units (a.u.) on the vertical axis. The horizontal axis denotes time since the start of meiosis II.

Figure S4 (related to Figure 3E). Dynamics for all exit network elements in *clb4Δ* cells, with the concentration of Clb4 set to zero. Concentrations are in arbitrary units (a.u.) on the vertical axis. The horizontal axis denotes time since the start of meiosis II.

Figure S5 (related to Figure 3F). Dynamics for all exit network elements in *clb3Δ* cells, with the concentration of Clb3 set to zero. Concentrations are in arbitrary units (a.u.) on the vertical axis. The horizontal axis denotes time since the start of meiosis II.

Figure S6 (related to Figure 4B). Dynamics for all exit network elements in *rim4-47AΔ* cells, with delayed Rim4 degradation. Concentrations are in arbitrary units (a.u.) on the vertical axis. The horizontal axis denotes time since the start of meiosis II.

Figure S7 (related to Figure 5A). Dynamics for all exit network elements in *ama1Δ* cells, with the concentration of Ama1 set to zero. Concentrations are in arbitrary units (a.u.) on the vertical axis. The horizontal axis denotes time since the start of meiosis II.

Figure S8. Single ClbT species. Clb1, Clb4, and Clb3 are replaced by a single, total Clb variable in the model, ClbT, with kinetic parameters given by that of Clb1. We note that the total Clb concentration drops below threshold at tthreshold = 24.3 min, corresponding to a faster exit from meiosis II (compared to the experimentally observed 38 ±18 min). Since the values of kinetic parameters for Clb1 and Clb4 are comparable, the dynamics shown here is similar to the Clb3 mutant (Fig. S5). The later expression of Clb3 upon Rim4 release, even at modest concentrations relative to Clb1 and Clb4, serves to maintain higher levels of total Cdk1-Clb kinase activity for longer, thereby prolonging exit.

Figure S9. mRNA-Rim4 dissociation and Rim4 clearance given by $f(t;\tau_p,T)$ (SI Eq. 1). Red: T = 20 min, τ_p^{-1} = 0.2 min^{-1} (WT). Purple: T = 28 min, τ_p^{-1}= 0.2 min^{-1} (*rim4-20A*). Green: T = 34 min, τ_p^{-1}= 0.2 min^{-1} (*rim4-27A*). Blue: T = 38 min, τ_p^{-1}= 0.2 min^{-1} (*rim4-47A-1*). Yellow: T = 44 min, τ_p^{-1}= 0.08 min^{-1} (*rim4-47A-2*). To describe Rim4 phosphorylation mutants, the onset time, T, and rate of mRNA release (and Rim4 clearance), τ_p^{-1}, can plausibly change with respect to wild-type. The horizontal axis is time in meiosis II, and on the vertical axis, a value of 1.0 corresponds to complete sequestration of mRNA by Rim4, and 0.0 corresponds to complete release of mRNA and Rim4 clearance.

Figure S10. Total Cdk1-Clb concentration for WT and different Rim4 mutants. Red: WT (T = 20 min, τ_p^{-1} = 0.2 min^{-1}). Purple: *rim4-20A* (T = 28 min, τ_p^{-1} = 0.2 min^{-1}). Green: *rim4-27A* (T = 34 min, τ_p^{-1}= 0.2 min^{-1}). Blue: *rim4-47A-1* (T = 38 min, τ_p^{-1}= 0.2 min^{-1}). Orange: *rim4-47A-2* (T = 44 min, τ_p^{-1}= 0.08 min^{-1}). Dashed red: Total Cdk1-Clb threshold. We note that with increasing T, and therefore delay in dissociation of mRNA from Rim4 (and Rim4 clearance), threshold crossing is delayed, until oscillatory dynamics sets in.
